# ARGONAUTE2 Localizes to Sites of Sporocysts in the Schistosome-Infected Snail, *Biomphalaria glabrata*

**DOI:** 10.3390/genes15081023

**Published:** 2024-08-03

**Authors:** Phong Phan, Conor E. Fogarty, Andrew L. Eamens, Mary G. Duke, Donald P. McManus, Tianfang Wang, Scott F. Cummins

**Affiliations:** 1 Centre for Bioinnovation, University of the Sunshine Coast, Maroochydore, QLD 4558, Australia; tuanphong.phan@research.usc.edu.au (P.P.); conor.fogarty@research.usc.edu.au (C.E.F.); twang@usc.edu.au (T.W.); 2 School of Science, Technology and Engineering, University of the Sunshine Coast, Maroochydore, QLD 4558, Australia; 3 School of Health, University of the Sunshine Coast, Maroochydore, QLD 4558, Australia; aeamens@usc.edu.au; 4 Molecular Parasitology Laboratory, QIMR Berghofer Medical Research Institute, Brisbane, QLD 4006, Australia; mary.duke@qimrberghofer.edu.au

**Keywords:** *Biomphalaria glabrata*, *Schistosoma mansoni*, host–parasite interaction, microRNA (miRNA) pathway, miRNA pathway protein machinery, Argonaute2 (Ago2), miRNA-directed gene expression regulation, immune response

## Abstract

MicroRNAs (miRNAs) are a class of small regulatory RNA that are generated via core protein machinery. The miRNAs direct gene-silencing mechanisms to mediate an essential role in gene expression regulation. In mollusks, miRNAs have been demonstrated to be required to regulate gene expression in various biological processes, including normal development, immune responses, reproduction, and stress adaptation. In this study, we aimed to establishment the requirement of the miRNA pathway as part of the molecular response of exposure of *Biomphalaria glabrata* (snail host) to *Schistosoma mansoni* (trematode parasite). Initially, the core pieces of miRNA pathway protein machinery, i.e., Drosha, DGCR8, Exportin-5, Ran, and Dicer, together with the central RNA-induced silencing complex (RISC) effector protein Argonaute2 (Ago2) were elucidated from the *B. glabrata* genome. Following exposure of *B. glabrata* to *S. mansoni* miracidia, we identified significant expression up-regulation of all identified pieces of miRNA pathway protein machinery, except for Exportin-5, at 16 h post exposure. For Ago2, we went on to show that the *Bgl*-Ago2 protein was localized to regions surrounding the sporocysts in the digestive gland of infected snails 20 days post parasite exposure. In addition to documenting elevated miRNA pathway protein machinery expression at the early post-exposure time point, a total of 13 known *B. glabrata* miRNAs were significantly differentially expressed. Of these thirteen *B. glabrata* miRNAs responsive to *S. mansoni* miracidia exposure, five were significantly reduced in their abundance, and correspondingly, these five miRNAs were determined to putatively target six genes with significantly elevated expression and that have been previously associated with immune responses in other animal species, including humans. In conclusion, this study demonstrates the central importance of a functional miRNA pathway in snails, which potentially forms a critical component of the immune response of snails to parasite exposure. Further, the data reported in this study provide additional evidence of the complexity of the molecular response of *B. glabrata* to *S. mansoni* infection: a molecular response that could be targeted in the future to overcome parasite infection and, in turn, human schistosomiasis.

## 1. Introduction

Mollusks represent the second-largest phylum of the animal kingdom, and member species of this phylum impact humans in various ways, from their traditional use in aquaculture through to their current application in healthcare and medicine. Yet, relatively little molluskan research has focused on the field of non-coding RNA (ncRNA) biology, highlighted by ncRNAs either deposited in the microRNA (miRNA) database, miRBase (www.mirbase.org), or reported in publications [1,2,3,4,5]. In mollusks, much of the research on ncRNA biology performed to date has focused on the miRNA class of small regulatory RNA. This work has included the identification of novel miRNAs, expression profiling of known miRNAs, functional characterization and comparative genomics studies of both novel and known miRNAs, environmental stress-responsive miRNA studies, and the documentation of the specific miRNA species involved in muscle development, nervous system regeneration, and immune responses [6,7,8,9,10,11].

Regarding the immune response, the freshwater snail *Biomphalaria* has been of most interest due to its role as an intermediate host for parasitic flatworms of the genus *Schistosoma*, which cause schistosomiasis in humans. Thus, increasing research on the genetics and genomics of *Biomphalaria* species, including efforts to understand their immune response to *Schistosoma* infection, has been performed. Towards this end, and to uncover the miRNA landscape of *Biomphalaria glabrata* (*B. glabrata*), a total of 107 novel precursor miRNAs (pre-miRNAs; stem-loop-structured precursor transcripts from which mature miRNAs are processed) and 95 known pre-miRNAs were predicted from the *B. glabrata* genome [12]. Furthermore, 102 mature miRNAs were putatively determined to be generated from the 95 known pre-miRNAs identified in the *B. glabrata* genome post precursor transcript processing by the core protein machinery of the *B. glabrata* miRNA pathway, and of these, 36 miRNAs were confirmed using transcriptomics [12]. In comparison with another gastropod, *Lottia gigantea* (owl limpet), 53 miRNAs shared 90% nucleotide content, and in addition, 13 *B. glabrata* miRNAs were determined to have a highly conserved nucleotide sequence composition in comparison to known miRNAs of other animal species previously deposited into the miRBase database [12].

*Biomphalaria glabrata* miRNAs are distributed throughout its tissues, including the ovotestis, digestive gland, stomach, albumen gland, heart, terminal genitalia, mantle edge, kidney, head, foot, salivary glands, the nervous system, and buccal mass [12]. While certain miRNAs such as miR-96, which has been demonstrated previously to play a role in cancer cell proliferation and tumor metastasis in humans [13,14], were found to be present in all tissues of the snail [12], let-7, a miRNA required for cell proliferation in numerous animal species, including the model nematode *Caenorhabditis elegans* [15], was found to be exclusively expressed in the albumen gland. While progress has been made in understanding miRNAs in *Biomphalaria*, further research is required to elucidate their functional importance and regulatory mechanisms, including the potential roles mediated by miRNAs in host–parasite interactions. Further discovery of the roles of miRNAs in *Biomphalaria* will not only provide valuable insights into the biology of this species and of other snail species but could potentially provide crucial information for use in the future development of novel strategies for controlling schistosomiasis transmission.

In this study, we documented the expression profile of core protein machinery of the *B. glabrata* miRNA pathway, including Drosha, DGCR8, Exportin-5, Ran, Dicer, and Ago2. We subsequently show that the Ago2 protein was expressed in all assessed *B. glabrata* tissues, a finding that confirms the importance of Ago2, the functional core of the miRNA-induced silencing complex (miRISC), which uses loaded miRNAs to direct expression regulation of target genes. Furthermore, we went on to show that 20 days post exposure to *S. mansoni* miracidia, the Ago2 protein was preferentially localized to the tissues that surround the sporocysts, which formed in the digestive gland of infected snails: a finding that suggests that miRISC (i.e., Ago2)-mediated gene expression regulation is required as part of the immune response of *B. glabrata* to *S. mansoni* infection. Further support that miRNA-directed gene expression regulation forms part of the immune response of *B. glabrata* to *S. mansoni* infection was our discovery of significantly altered abundance in infected snails for five *B. glabrata* miRNAs that putatively target six genes previously associated with immune responses in other animal species, including humans. Taken together, the findings presented in this study not only provide additional evidence of the complexity of the molecular response of *B. glabrata* to *S. mansoni* infection but could potentially provide new avenues that can be targeted in the future to overcome intermediate parasite infection and, in turn, human schistosomiasis.

## 2. Materials and Methods

### 2.1. Biomphalaria glabrata and Schistosoma mansoni Maintenance

*Schistosoma mansoni* parasites were maintained under an Australian Department of Agriculture, Fisheries and Forestry Biosecurity permit, with all associated animal experimentation conducted in strict compliance with approved ethical protocols. The procedures involving animal models received prior approval from the Animal Ethics Committee of the QIMR Berghofer Medical Research Institute (QIMR-B), Brisbane (Project number: P3705). The *B. glabrata* snails (NMRI strain) were reared in calcium carbonate (CaCO_3_)-conditioned water (pH 7.0) under aerated conditions at a constant temperature of 27 °C, and day/night lighting cycle of 12 h (h) of light and 12 h of dark. The diet fed to the *B. glabrata* snails consisted of algal tablets and lettuce thoroughly washed with reverse osmosis water to prevent the introduction of extraneous particulates. In addition, the Swiss mice that were utilized in this study as definitive hosts were maintained in a quarantine containment area within a Specific Pathogen Free Animal Facility under Biosecurity Quarantine Control. All in vivo studies were performed in accordance with the Guide for the Care and Use of Laboratory Animals stipulated by the National Institutes of Health.

### 2.2. MicroRNA Pathway Gene Identification in Biomphalaria glabrata

Initially, the *B. glabrata* Drosha, DiGeorge critical region-8 (DGCR8), Exportin-5 (Exp5), RAS-related nuclear protein (Ran), Dicer (Dcr), and Argonaute2 (Ago2) protein sequences were identified via the use of the human (*Homo sapiens*) homologous protein sequences as “*bait*” in a standard BLASTp search of the NCBI non-redundant protein database. For each performed search, the retrieved result that also returned the lowest E-value was selected for use to represent the *B. glabrata* homolog of each piece of protein machinery known to form the functional core of the human miRNA pathway. Functional domains of the identified proteins, including, as an example, the amino (N)-terminal domain, ribonuclease III carboxyl terminal (RIBOc) domain, double-stranded RNA-binding motif (DSRM), PIWI, Argonaute and Zwille (PAZ) domain, and P-element-induced wimpy testis (PIWI) domain, among others, were then annotated onto the identified amino acid backbone sequences by use of the Simple Modular Architecture Research Tool [16].

### 2.3. Differential Expression of miRNA Pathway Genes in Biomphalaria glabrata Following Its Exposure to Schistosoma mansoni Miracidia

Non-infected *B. glabrata* individuals (controls) and those that had been exposed to *S. mansoni* miracidia 16 h and 42 h prior to sample collection were harvested in triplicates at the QIMR-B culturing facility and stored in RNAlater solution (Thermo Fisher Scientific, Waltham, MA, USA). The isolation of total RNA was performed using TRIzol Reagent according to the manufacturer’s protocol (Thermo Fisher Scientific). After extraction and treatment with DNase I to remove any contaminating genomic DNA, the total RNA fraction was resuspended in RNase-free water, and the quality and quantity of each sample were determined on a Nanodrop ND-2000 (Thermo Fisher Scientific) spectrophotometer using absorbance wavelengths of 260 nanometers (nm) and 280 nm to determine the 260/280 nm (A_260_/A_280_) absorbance ratio. Only total RNA samples determined to be of high quality and purity were sent to Novogene (Hong Kong, China) for next-generation RNA sequencing (RNA-Seq) on an Illumina 2500 platform (Gene Expression Omnibus (GEO) Database accession numbers: SRR18863902, SRR28266338, and SRR28266337). Genome-guided mapping was carried out using the *B. glabrata* reference genome retrieved from VectorBase (BglabrataBB02) using the CLCbio Genomics Workbench (Qiagen, Hilden, Germany). The number of reads as determined by transcripts per million (TPM) was obtained as according to the methods outlined in [16]. Finally, *Drosha*, *DGCR8*, *Exp5*, *Ran*, *Dcr*, and *Ago2* gene expression was determined, and a Student’s *t*-test was then used to determine statistical significance (*p*-value < 0.05).

### 2.4. Production of a Polyclonal Antibody Specific to Biomphalaria glabrata Ago2

A polyclonal antibody specific to the *B. glabrata* Ago2 protein (*Bgl*-Ago2) was produced in two male New Zealand white rabbits by Genscript (Piscataway, NJ, USA) using their established protocols. First, a synthetic ELEVTLPGEGRDRVF peptide, which forms an encoded fragment of the PAZ domain of *Bgl*-Ago2, was generated and conjugated to bovine serum albumin (BSA) via treatment with the condensing agent N-(3-dimethylaminopropyl)-N′-ethylcarbodiimide hydrochloride (EDAC HCl). For immunization, the rabbits were subcutaneously injected with the *Bgl*-Ago2 fusion protein after its mixing with an equal volume of complete Freund’s adjuvant. Immunization of the two male New Zealand white rabbits was performed at 2-week intervals. Rabbit blood was collected 2 weeks after the last immunization, incubated at 37 °C for 2 h to allow clotting, and then centrifuged at 2000× *g* for 10 min at room temperature to collect the serum. The collected serum was affinity purified and then stored at −20 °C until required for use in Western blot hybridization analysis and immunofluorescence histology.

### 2.5. Western Blot Hybridization Analysis

Non-infected and infected (20 days post exposure) *B. glabrata* were collected from the QIMR-B culturing facility. The whole tissue (minus the shells) was homogenized in buffered solution (2.0% (*w*/*v*) sodium dodecyl sulfate (SDS); 1 × phosphate-buffered saline (PBS); 10 nM of β-mercaptoethanol) and centrifuged at 12,000 × *g* for 3 min at room temperature. The resulting supernatant was collected, and the total protein concentration determined in a spectrophotometer via measurement at an absorbance wavelength of 280 nm. Isolation of *S. mansoni* miracidia at ~2 h post hatching was conducted as described previously [17], and total protein was extracted using the same method as outlined for *B. glabrata* whole tissues. Approximately 7.5 micrograms (µg) of total protein, along with a molecular weight marker (Bio-Rad, Hercules, CA, USA), was separated using a Mini PROTEAN precast gel (Bio-Rad) via standard electrophoresis at 150 volts (V). After electrophoresis, the separated protein was transferred onto a positively charged 0.2 micrometer (μm) PVDF membrane (Bio-Rad) using a Trans-Blot Turbo Transfer System (Bio-Rad) as per the manufacturer’s instructions. After total protein transfer, the membrane was blocked in a 4.0% blocking solution (5.0% (*w*/*v*) skim milk powder; 1 × PBS; 0.1% (*v*/*v*) Tween-20) at room temperature for 1 h. The blocked membrane was then incubated overnight at 4 °C in a primary antibody solution that consisted of the rabbit anti-*Bgl*-Ago2 polyclonal antibody diluted 1:500 in 1 × PBS supplemented with 0.1% (*v*/*v*) Tween-20 (PBST). The membrane was washed in multiple changes of fresh PBST, and then, the secondary antibody (1:10,000 rabbit anti-Ig-IR 680, LI-COR, Lincoln, NE, USA) was added to a fresh change of PBST, and the membrane was incubated at room temperature for 1 h. Following another series of washes using fresh changes of PBST, antibody binding to the probed membrane was detected using an Odyssey CLx *Chemiluminescent* Imaging System (LI-COR).

### 2.6. Histology and Immunofluoresence to Determine Ago2 Localisation in Biomphalaria glabrata Tissues Following Its Exposure to Schistosoma mansoni Miracidia

The whole bodies of non-infected (control) and infected (20 days post exposure) *B. glabrata* were removed from their shells and washed twice in fresh changes of Milli-Q water, before being fixed in 4.0% (*w*/*v*) paraformaldehyde (pH 7.4) for 10 h at 4.0 °C. The fixed samples were washed three times in fresh changes of 70% ethanol and then dehydrated through a graded series of ethanol solutions (70%, 80, 90%, and 100%) until the 100% ethanol solution was reached. Following two 30 min room temperature incubations in xylene, the samples were incubated in wax (Histoplast PE, Thermo Fisher Scientific), and then embedded in paraffin. Sections were generated using a Leica microtome set at an 8 µm cutting distance, and the resulting sections were transferred onto Superfrost microscope slides (Thermo Fisher Scientific).

For histology, hematoxylin and eosin (H&E) staining was completed via following a standard staining protocol [18]. For mounting, sections were cleared using xylene solution, and then, a coverslip was applied with dibutylphthalate polystyrene xylene-based mounting medium. Slides were viewed under a Leica DM550 microscope equipped with a charge-coupled device camera.

For immunofluorescence, slides were deparaffinized and then rehydrated through an ethanol dilution series. After washing three times with fresh changes of 0.1% (*v*/*v*) PBST, sections were incubated at room temperature for 2 h in 4.0% (*v*/*v*) normal goat serum (NGS) in PBST. Sections were again washed using three changes of fresh 0.1% PBST, and then, the washed sections were incubated overnight at 4 °C in fresh 4.0% NGS in PBST that had also been supplemented with the anti-*Bgl*-Ago2 primary antibody at a 1:200 dilution. Following the overnight incubation, slides were washed three times in fresh changes of PBST, and then, the slides were incubated in the dark for 2 h at room temperature in fresh 4.0% NGS in PBST that had been supplemented with the goat anti-rabbit Alexa Fluor 488 IgG secondary antibody (ThermoFisher, #A-11008) at a 1:500 dilution. After secondary antibody incubation, the sections were washed three times in fresh changes of PBST, and then, the washed sections were mounted in 10% (*v*/*v*) glycerol diluted in 1 × PBS. As a negative control, tissue sections were incubated with 4.0% NGS that had been freshly diluted in PBST instead of probing with the anti-*Bgl*-Ago2 primary antibody. However, all other steps in the preparation of the control sections were as per outlined for the experimental samples. Finally, the control and infected sample sections were viewed via microscopy using a Leica DM550 fluorescence microscope equipped with a Leica camera.

### 2.7. High-Throughput Small RNA Sequencing for Profiling of the microRNA Landscape of Biomphalaria glabrata Post Exposure to Schistosoma mansoni Miracidia

As reported for the high-molecular-weight RNA (i.e., messenger RNA (mRNA)) analysis, non-infected *B. glabrata* individuals (controls) and those that had been exposed to *S. mansoni* miracidia 16 h prior to sample collection were harvested in triplicates at the QIMR-B culturing facility and stored in RNAlater solution (Thermo Fisher Scientific). The isolation of total RNA was performed using TRIzol Reagent according to the manufacturer’s protocol (Thermo Fisher Scientific), and all total RNA extractions were subsequently treated with DNase I to remove any contaminating genomic DNA. After DNase I treatment, the DNA-free total RNA samples were resuspended in RNase-free water, and the quality and quantity of each sample were determined on a Nanodrop ND-2000 (Thermo Fisher Scientific) spectrophotometer using absorbance wavelengths of 260 and 280 nm to determine the A_260_/A_280_ ratio. Only total RNA samples determined to be of high quality and purity were sent to Novogene (Hong Kong) for next-generation RNA sequencing of the small fraction (sRNA-Seq) on an Illumina NovaSeq SE50 platform (GEO Database accession numbers: SRR28254504, SRR28254505, and SRR28254503). The sRNA reads were filtered according to the quality scores obtained from use of the FastQC software, and for each high-quality sRNA read, the adapter sequences were next trimmed via the use of the Trimmomatic software [18]. Finally, each sRNA read with a sequence length of between 18 to 35 nucleotides was retained, and then, each of the remaining sRNA reads within this size class was again assessed for its quality. Following this analysis, all reads deemed to be of low quality (i.e., those that returned a quality score of <25) were discarded to exclude their further use in this study.

All valid sRNA reads were counted and mapped onto the *B. glabrata* genome derived from VectorBase via use of the Bowtie and miRDeep2 software [19]. Furthermore, both the *B. glabrata* pre-miRNA and mature miRNA sequences that were generated for use in a previous study [12] were used as a reference source for the sRNA analyses performed in this study. Although both novel and known *B. glabrata* miRNAs were identified previously [20], only the expression level of known miRNAs was used here to compare the miRNA profile of *S. mansoni* miracidia-infected *B. glabrata* to the miRNA profile of non-infected control *B. glabrata* animals.

For the *B. glabrata* miRNA target gene identification analysis, the 3′ untranslated region (3′ UTR) was extracted from the annotation of genic features defined in the GFF3 file from the *B. glabrata* genome, and each 3′ UTR was assessed for its “*likelihood*” to form a *B. glabrata* miRNA target gene by use of the software RNAhybrid [21] and miRanda [22]. In both the RNAhybrid and miRanda analyses, parameters were set at (1) a maximum threshold free energy of equal to or less than −20 kcal/mol and (2) a *p*-value less than or equal to 0.05. Following the establishment of these initial parameters, putative target genes were only considered further if the 3′ UTR transcript and the binding miRNA could complement one another perfectly (i.e., form a double-stranded RNA (dsRNA) hybrid) along the entire length of the seed region of the target site of the miRNA target gene and with the formed dsRNA hybrid returning a minimum free energy score of equal to or less than −10 kcal/mol.

## 3. Results

### 3.1. Identification, Annotation, and Expression Analysis of Core Pieces of Protein Machinery of the Biomphalaria glabrata miRNA Pathway

**Figure 1:**
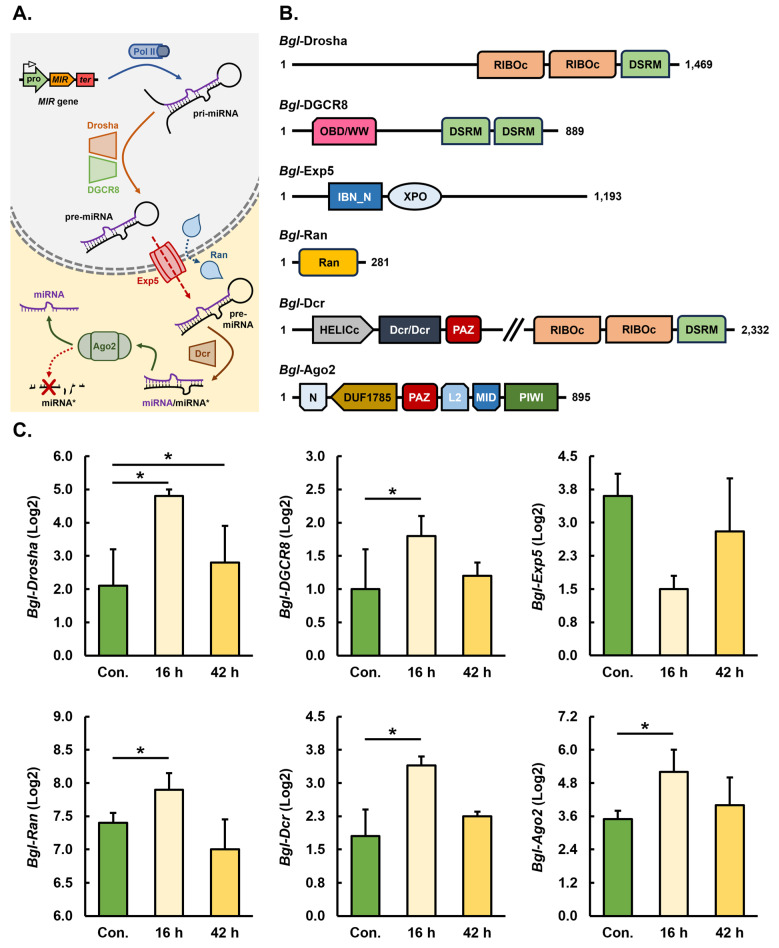
Identification and expression analysis of core pieces of protein machinery of the *Biomphalaria glabrata* miRNA pathway 16 and 42 h after its exposure to *Schistosoma mansoni* miracidia. (**A**) Schematic of the production (nucleus; grey-colored shaded region) and action (cytoplasm; pale-yellow-colored shaded region) stages of the *B. glabrata* miRNA pathway with the functional position within the pathway of *Bgl*-Drosha, *Bgl*-DGCR8, *Bgl*-Exp5, *Bgl*-Ran, *Bgl*-Dcr, and *Bgl*-Ago2 indicated. (**B**) Schematic outlining the functional domain structure of the core pieces of protein machinery of the *B. glabrata* miRNA pathway, including *Bgl*-Drosha, *Bgl*-DGCR8, *Bgl*-Exp5, *Bgl*-Ran, *Bgl*-Dcr, and *Bgl*-Ago2. RIBOc, ribonuclease III C terminal domain; DSRM, double-stranded RNA motif; OBD/WW, origin-binding domain/tryptophan-tryptophan domain; XPO, Exportin domain; IBN_N, Importin-β N-terminal domain; Ran, Ras-related nuclear domain; Dcr/Dcr, Dicer dimerization domain, HELICc, helicase superfamily C-terminal domain; PAZ, PIWI, Argonaute, and Zwille domain; N, Ago protein amino-terminal region domain; DUF1785, domain of unknown function 1785 domain; PIWI, P-element-induced wimpy testis domain; L2, linker region 2; MID, middle domain. (**C**) RNA-Seq assessment of the altered expression of *Bgl-Drosha*, *Bgl-DGCR8*, *Bgl-Exp5*, *Bgl-Ran*, *Bgl-Dcr*, and *Bgl-Ago2*, 16 and 42 h after exposure of *B. glabrata* animals to the *S. mansoni* parasite. Error bars represent the standard error of the mean, and an asterisk (*) denotes significantly altered transcript abundance (*p*-value ≤ 0.05) at either the 16 or 42 h time point compared to the level of expression of each core piece of protein machinery in control (unexposed) *B. glabrata* animals.

Considering that the presence of both novel and conserved miRNAs was reported previously for *B. glabrata* [12], we initially sought to determine the degree of conservation of core pieces of protein machinery of the *B. glabrata* miRNA pathway. As depicted in the Figure 1A schematic, two pieces of protein machinery per stage of the *B. glabrata* miRNA pathway were selected for further analysis to uncover the degree of functional conservation of the miRNA pathway in *B. glabrata*. More specifically, the six pieces of protein machinery selected for this initial analysis included (1) the nucleus-localized microprocessor core components, Drosha and its functional partner protein DiGeorge critical region-8 (DGCR8), (2) the pre-miRNA nucleus exportation proteins Exportin-5 (Exp5) and RAS-related nuclear protein (Ran), and the (3) cytoplasm-localized pre-miRNA processing protein Dicer (Dcr) and the catalytic core of the miRNA-directed RNA-induced silencing complex (miRISC), Argonaute2 (Ago2) (Figure 1A).

For the initial identification of *B. glabrata* miRNA pathway protein machinery (prefixed by *Bgl*), the human homologous protein (prefixed by *Hsa*) was used as the “*bait*” in a standard BLASTp search of the NCBI non-redundant protein database. A single *Hsa*-Drosha homolog was identified for *B. glabrata* via BLASTp searching of the NCBI non-redundant protein database, with the sequence of the identified *Bgl*-Drosha candidate protein revealed to be 1469 amino acid residues in length (Figure S1A). Annotation of the *Bgl*-Drosha candidate sequence further showed the presence of two ribonuclease III (RIBOc) domains and a single dsRNA binding motif (DSRM) proximally positioned together in the C-terminal region of the identified protein sequence (Figure 1B). The annotation of the identified *B. glabrata* protein sequence strongly inferred that the correct *Bgl*-Drosha protein homolog of *Hsa*-Drosha had been successfully identified.

As for other dsRNA-binding (DRB) proteins involved in RNA-silencing pathways, the identified *Bgl*-DGCR8 homolog following BLASTp searching of the non-redundant protein database via the use of the *Hsa*-DGCR8 protein sequence as bait was revealed to be considerably smaller in size at 889 amino acid residues compared to its partnering endonuclease, *Bgl*-Drosha (Figure S1B). Furthermore, annotation of the sequence of the *Bgl*-DGCR8 candidate protein identified the presence of two C-terminus-localized DSRMs and a single origin-binding domain (OBD) or WW (tryptophan-tryptophan) domain located in the amino-terminal half of the protein (Figure 1B).

A *B. glabrata* homolog of *Hsa*-Exp5 was next identified by our initial analysis, with the full-length sequence of the identified *Bgl*-Exp5 candidate protein consisting of 1193 amino acids (Figure S1C). Subsequently, the amino acid sequence of the identified *Bgl*-Exp5 candidate protein was subsequently determined to encode an Exportin (XPO) domain and an Importin-β N-terminal (IBN_N) domain at its N-terminus (Figure 1B). Confirmation of the presence of both the XPO and IBN_N domains in the identified *Bgl*-Exp5 candidate protein, together with the similar N-terminal positioning of these two functional domains in the *Bgl*-Exp5 and *Hsa*-Exp5 homologs, strongly implies that the identified *Bgl*-Exp5 candidate protein would perform a similar pre-miRNA nucleus exportation role in the *B. glabrata* miRNA pathway (Figure 1A) as Hsa-Exp5 in the human miRNA pathway. Again, via the use of the human Ran protein (*Hsa*-Ran) sequence as bait, BLASTp searching of the NCBI non-redundant protein database allowed for the identification of a strong candidate Ran protein homolog for *B. glabrata*. Namely, a small 281 amino acid protein sequence (Figure S1D) that was determined to only encode a single Ras-related nuclear (Ran) domain across the majority of the amino acid residues of the protein, specifically residues 15 to 216, was identified (Figure 1B).

Use of the *Hsa*-Dcr sequence in our BLAST search analyses of the *B. glabrata* proteome, identified a long-length sequence composed of 2332 amino acid residues (Figure S1E). Annotation of the functional domain landscape of the identified sequence revealed the presence of a helicase superfamily C-terminal (HELICc) domain encoded by residues 481 to 574 and a PIWI, Argonaute and Zwille (PAZ) domain across residues 930 to 1100 in the N-terminal half of the protein (Figure 1B). In addition, two RIBOc domains at residues 1796 to 2012 and 2079 to 2258, respectively, and a single DSRM encoded by residues 2261 to 2324 were positioned proximally to each other in the C-terminal half of the identified *B. glabrata* sequence (Figure 1B). The presence of HELICc, PAZ, and RIBOc domains and a DSRM in the annotated sequence strongly inferred that the identified protein was indeed the cytoplasm-localized miRNA pathway endonuclease *Bgl*-Dcr, the *B. glabrata* homolog of the *Hsa*-Dcr protein used as bait in the BLASTp search.

Furthermore, protein homology searching of the *B. glabrata* proteome using the *Hsa*-Ago2 sequence as bait identified a candidate protein 895 amino acid residues in length (Figure S1F). Functional domain annotation of the putative *Bgl*-Ago2 candidate sequence revealed a conserved N-terminal region (N) domain (an Ago protein family member specific domain), a domain of unknown function 1785 (DUF1785) domain, a PAZ domain, a conserved linker region (L2) between the PAZ and Middle (MID) domains, and a C-terminal half-positioned P-element-induced wimpy testis (PIWI) domain (Figure 1B). This evidence that the sequence of the putative *Bgl*-Ago2 candidate protein identified here encodes all domains essential for the conserved functional roles demonstrated for characterized members of the Ago protein family strongly indicates that the identified protein was indeed *Bgl*-Ago2, the *B. glabrata* homolog of *Hsa*-Ago2.

Following the identification of a strong candidate protein representative for the *B. glabrata* miRNA pathway protein machinery *Bgl*-Drosha, *Bgl*-DGCR8, *Bgl*-Exp5, *Bgl*-Ran, *Bgl*-Dcr, and *Bgl*-Ago2, the level of expression of the encoding gene for each of these six pieces of core protein machinery of the miRNA pathway was assessed following the exposure of *B. glabrata* to *S. mansoni* miracidia at the 16 and 42 h post-exposure time points. Analysis of the expression trends of *Bgl*-*Drosha*, *Bgl*-*DGCR8*, *Bgl*-*Exp5*, *Bgl*-*Ran*, *Bgl*-*Dcr*, and *Bgl*-*Ago2* was also performed via this RNA-Seq approach on non-exposed *B. glabrata* individuals (i.e., control animals) to uncover any “*shared*” or “*global*” trends in gene expression to attempt to determine the extent of involvement of the miRNA pathway as part of the molecular response of *B. glabrata* to *S. mansoni* miracidia exposure. At 16 h post exposure to *S. mansoni* miracidia, the expression of the *Bgl-Drosha*, *Bgl-DGCR8*, *Bgl-Ran*, *Bgl-Dicer,* and *Bgl-Ago5* genes was determined by RNA-Seq to be significantly elevated (Figure 1C). In comparison to the 16 h post-exposure time point, the expression of *Bgl-Drosha*, *Bgl-DGCR8*, *Bgl-Dcr,* and *Bgl-Ago2* only remained mildly elevated, compared to the control, at the 42 h post-exposure time point (Figure 1C). In contrast to the maintained elevated level of expression of *Bgl-Drosha*, *Bgl-DGCR8*, *Bgl-Dcr,* and *Bgl-Ago2* at 42 h post exposure to *S. mansoni* miracidia, the expression of *Bgl-Ran* was reduced to a degree lower than the level of *Bgl-Ran* gene expression in control *B. glabrata* animals (Figure 1C). In addition, and at both the 16 and 42 h exposure time points, the expression level of *Bgl-Exp5* was revealed by RNA-Seq to be significantly reduced at 16 h post exposure and to remain moderately reduced at the 42 h post-exposure time point (Figure 1C). In spite of the observed expression trends for the *Bgl-Ran* (at the 42 h time point) and *Bgl-Exp5* genes (at the 16 and 42 h time points), the overall promotion of gene expression of the analyzed core pieces of protein machinery of the *B. glabrata* miRNA pathway strongly inferred that the miRNA pathway directs a primary molecular response, at least during the initial stage of exposure of *B. glabrata* to *S. mansoni* miracidia (Figure 1C).

### 3.2. Expression Analysis and Tissue Localization of the Biomphalaria glabrata Ago2 Protein Post Long-Term Infection by Schistosoma mansoni

**Figure 2:**
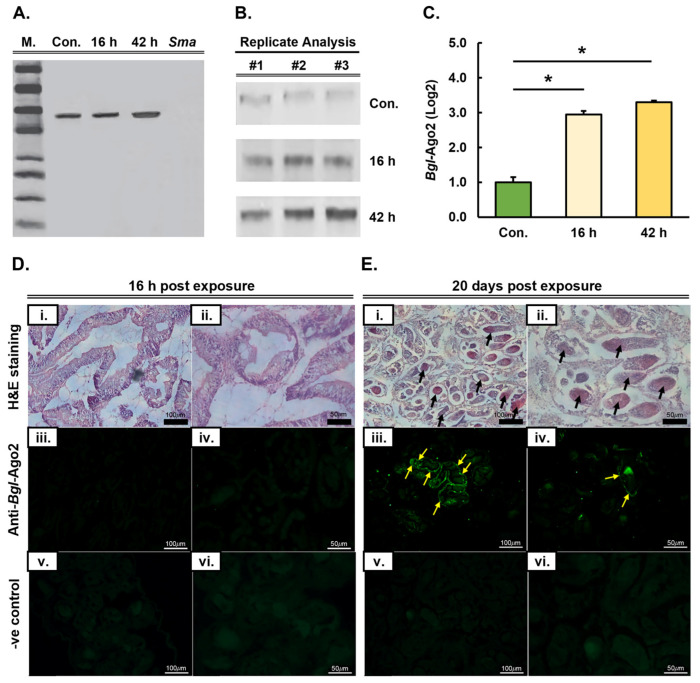
Expression analysis and tissue localization of the Ago2 protein in *Biomphalaria glabrata* animals following their short- and long-term exposure to the *Schistosoma mansoni* parasite. (**A**) Western blot hybridization analysis of Ago2 protein abundance in whole-protein extracts from unexposed (control) and exposed *B. glabrata* whole animals and *S. mansoni* miracidia. (**B**) Western blot hybridization analysis of Ago2 protein abundance in unexposed *B. glabrata* whole animals (control) and in exposed animals 16 and 42 h after their exposure to *S. mansoni* miracidia, an analysis that was performed in triplicate for quantification (**C**) to definitively demonstrate significantly altered Ago2 protein abundance in *S. mansoni*-exposed *B. glabrata* whole animals. An asterisk (*) denotes significantly altered Ago2 protein abundance (*p*-value ≤ 0.05) at either the 16 or 42 h time point compared to the level of Ago2 protein in control (unexposed) *B. glabrata* animals. (**D**) Light-field and fluorescent microscopic analysis of the intestinal digestive gland of sectioned *B. glabrata* animals 16 h post exposure to *S. mansoni* miracidia. Specifically, (**Di**,**Dii**) show different magnifications of bright-field microscopic analysis of H&E-stained regions of the *B. glabrate* intestinal digestive gland. No fluorescence was observed in the intestinal digestive gland of sectioned *B. glabrata* animals 16 h post exposure to *S. mansoni* miracidia (**Diii**,**Div**), nor were fluorescent signals observed in negative control samples (**Dv**,**Dvi**). (**E**) Light-field microscopic analysis and fluorescent microscopic analysis of the intestinal digestive gland of sectioned *B. glabrata* animals 20 days post exposure to the *S. mansoni* parasite. Specifically, (**Ei**,**Eii**) show different magnifications of bright-field microscopic analysis of H&E-stained regions of the intestinal digestive gland of sectioned *B. glabrate* animals, with the black arrows indicating sporocysts. Readily observable fluorescence was observed in the intestinal digestive gland of sectioned *B. glabrata* animals (yellow arrows), specifically around the sporocysts that had formed at this long-term exposure time point (**Eiii**,**Eiv**). As shown in (**Dv**,**Dvi**), no fluorescence was observed in the intestinal digestive gland sectioned of *B. glabrata* animals 20 days post exposure to *S. mansoni* miracidia in the negative control samples (**Eiv**,**Ev**).

Due to its central role in the miRNA pathway as the functional core of miRISC [23,24], together with our gene expression analysis revealing *Bgl-Ago2* expression to be elevated at both the 16 and 42 h time points post exposure to *S. mansoni* miracidia (Figure 1C), we next attempted to confirm the documented *Bgl-Ago2* gene expression changes at the protein level. Western blot hybridization analysis, using the total protein isolated from a single, unexposed control and miracidia-exposed *B. glabrata* whole-animal samples (minus their shells), was probed with an anti-*Bgl*-Ago2 antibody (Figure 2A). In the control, the unexposed *B. glabrata* individual, the presence of a single hybridization product at the expected size of ~93 kilodaltons (kDa) was observed (Figure 2A). In addition, the specificity of the antibody for *B. glabrata* Ago2 was confirmed by our inability to observe a hybridization product in the *S. mansoni* miracidia total protein extract included in this analysis as a negative control. In the exposed *B. glabrata* individual at both the 16 and 42 h time points, a single hybridization product was again observed (Figure 2A). Moreover, the single hybridization product observed in the 16 and 42 h post-exposure time point samples was the same size (~93 kDa) as the hybridization product observed in the control, a result that further confirmed the specificity of the anti-*Bgl*-Ago2 antibody that was generated for use in this study (Figure 2A).

Our initial Western blot hybridization analysis did, however, show that the *Bgl*-Ago2 protein accumulated to an equivalent level of abundance in the *B. glabrata* animal 16 h post exposure to *S. mansoni* miracidia, as was observed in the control, the unexposed *B. glabrata* individual. This formed an unexpected result, as our gene expression analyses via RNA-Seq (Figure 1C) indicated that *Bgl-Ago2* transcript abundance was elevated at both the 16 and 42 h time points. In accordance with the gene expression analyses, and when compared to the abundance of the *Bgl*-Ago2 protein in the control, our initial Western blot analysis did, however, show that the abundance of the *Bgl*-Ago2 protein was elevated in the *B. glabrata* individual 42 h post exposure to *S. mansoni* miracidia (Figure 2A). Therefore, having confirmed the specificity of the anti-*Bgl*-Ago2 antibody generated for use in this study, and to address the disparity in the gene expression analyses at the transcript and protein levels, we next repeated our Western blotting analysis of Ago2 protein abundance in unexposed and exposed *B. glabrata* individuals. Furthermore, this subsequent analysis was performed in triplicate to allow for the quantification of *Bgl*-Ago2 protein abundance in control and *S. mansoni* miracidia-exposed individuals. As shown in Figure 2B, when performed in triplicate, the Western blot hybridization analysis was in agreeance with our RNA-Seq result; that is, *Bgl*-Ago2 abundance was elevated in the *B. glabrata* individual 16 and 42 h post exposure to *S. mansoni* miracidia compared to the abundance of *Bgl*-Ago2 in the control animal. In addition, quantification of Ago2 protein abundance (Figure 2C) revealed that the degree of elevation was significant in the *B. glabrata* individual at both the 16 and 42 h post-exposure time points compared to the abundance of the *Bgl*-Ago2 protein in the control animal.

Following our confirmation that *Bgl*-Ago2 protein abundance was elevated in response to *B. glabrata* exposure to *S. mansoni* miracidia (Figure 2A–C), we sought to determine whether the increased level of Ago2 protein occurred in all *B. glabrata* tissues or if the abundance of *Bgl*-Ago2 was only elevated in a specific tissue. Towards this goal, whole *B. glabrata* body tissue was prepared from unexposed and exposed animals for histological examination via both standard and fluorescence microscopy. Two post-exposure time points were selected for inclusion in our histological analyses and included (1) the short-term exposure time point where whole-body tissue was sampled from *B. glabrata* animals 16 h after their exposure to *S. mansoni* miracidia and (2) the long-term time point where tissue was sampled from the whole body of a *B. glabrata* individual 20 days after its exposure to *S. mansoni* miracidia. Initially, histology was performed on whole-body samples of *B. glabrata* to determine the tissue localization of potential sporocysts that had formed either before or after exposure to *S. mansoni* miracidia (Figure S2). This histological analysis clearly showed that prior to exposing *B. glabrata* to *S. mansoni* miracidia, sporocysts were not present (Figure S2). Similarly, at the short-term time point, 16 h post exposure to *S. mansoni* miracidia, sporocysts were not visible in the intestinal digestive gland of *B. glabrata* (Figure 2D,E). In contrast to the pre-exposure and short-term exposure time point histological analyses (Figure 2D), sporocysts were readily visible in the intestinal digestive gland of *B. glabrata* individuals at the long-term exposure time point, 20 days post exposure to *S. mansoni* miracidia (Figure 2E). This observation readily demonstrated that the exposure of *B. glabrata* to *S. mansoni* miracidia had allowed the *S. mansoni* parasite to continue to progress through its developmental cycle in its intermediate host, *B. glabrata*.

Immunofluorescence microscopy was next used to determine *Bgl*-Ago2 protein localization in *B. glabrata* individuals 16 h and 20 days post exposure to *S. mansoni* miracidia. In accordance with our inability to observe sporocysts at the short-term exposure time point, no fluorescence post labeling with the Ago2-specific antibody could be observed via immunofluorescence microscopy 16 h post exposure (Figure 2D). At the long-term exposure time point, 20 days post exposure to *S. mansoni* miracidia, all of the fluorescent signals visualized were observed to be localized in close proximity to the sporocysts that had developed in the intestinal digestive gland of the exposed *B. glabrata* individual (Figure 2E). This finding strongly inferred that the elevated level of *B. glabrata* Ago2 protein abundance observed via Western blotting was due to highly localized accumulation of the Ago2 protein in *B. glabrata* tissues actively infected with the *S. mansoni* parasite. In addition, it is important to note here that no fluorescence was observed in the same regions at either the short- or long-term exposure time point in the negative control samples included in this analysis, which were not labeled with the *B. glabrata* Ago2-specific antibody (Figure 2D,E). However, some autofluorescence was observed within the radula and gizzard of negative control animals (Figure S3), with both tissue types known to contain high levels of chitin [25], the highly abundant polysaccharide of the mollusk exoskeleton.

### 3.3. Demonstration of the Requirement of the miRNA Pathway as Part of the Molecular Response of Biomphalaria glabrata to Exposure to Schistosoma mansoni Miracidia

The altered gene expression of *Bgl-Drosha*, *Bgl-DGCR8*, *Bgl-Ran*, *Bgl-Exp5*, *Bgl-Dcr,* and *Bgl-Ago2,* as revealed via our RNA-Seq analyses (Figure 1), together with localization of the *Bgl*-Ago2 protein to the sporocysts that formed in the *B. glabrata* intestinal digestive gland 20 days post exposure to *S. mansoni* miracidia (Figure 2), strongly inferred that the miRNA pathway plays a central role in the molecular response of *B. glabrata* to its infection by the *S. mansoni* parasite. Therefore, to further confirm the requirement of a miRNA-mediated molecular response to *S. mansoni* miracidia exposure in *B. glabrata*, changes in miRNA abundance were next investigated via a sRNA-Seq approach in *B. glabrata* whole-animal samples at the 16 h short-term post-exposure time point for comparison to unexposed control animals. The 16 h time point was selected for inclusion in this analysis over the 42 h time point based on our demonstration that *Bgl-Drosha*, *Bgl-DGCR8*, *Bgl-Ran*, *Bgl-Exp5*, *Bgl-Dcr,* and *Bgl-Ago2* gene expression was altered to a greater degree at this initially analyzed time point compared to the 42 h time point post exposure of *B. glabrata* to *S. mansoni* miracidia.

**Figure 3:**
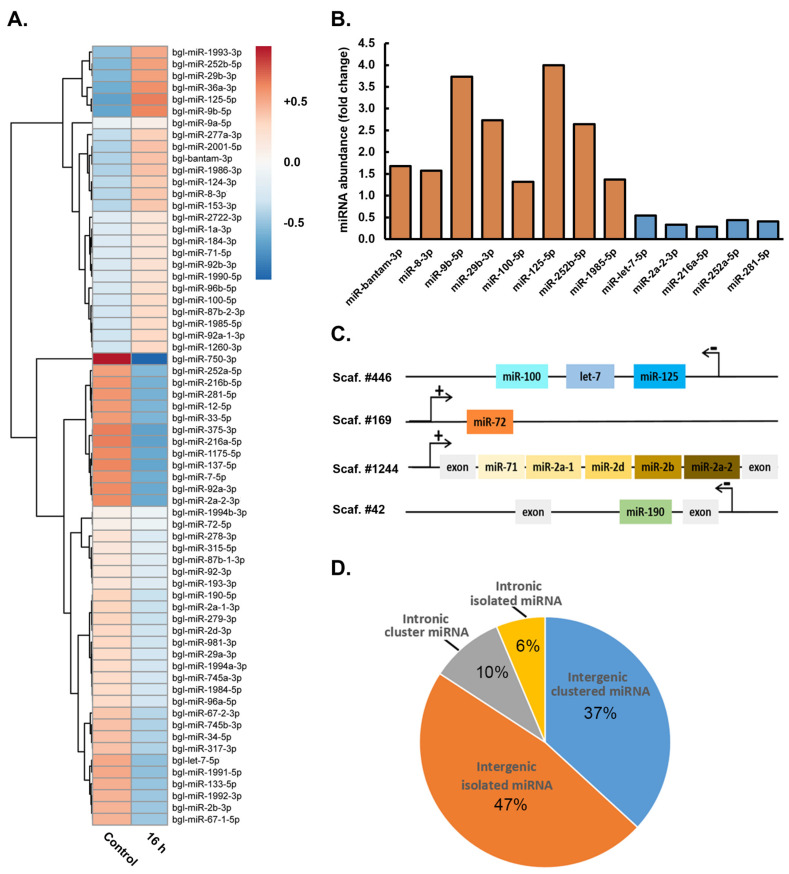
Profiling of the microRNA landscape of *Biomphalaria glabrata* whole animals 16 h after their exposure to *Schistosoma mansoni* miracidia. (**A**) Heat map of the 66 miRNAs with altered abundance (up- (orange) or down-regulated (blue) abundance) 16 h post exposure of *B. glabrata* to *S. mansoni* miracidia, with the intensity of shading of each tile indicating the degree of change to miRNA abundance. (**B**) Elevated (n = 8) or reduced (n = 5) levels of the 13 miRNAs with significantly altered abundance 16 h post exposure of *B. glabrata* to *S. mansoni* miracidia, with orange-colored columns showing up-regulated miRNAs and blue-colored columns representing down-regulated miRNAs. (**C**) Schematic demonstrating that miRNA abundance was altered in *B. glabrata* whole animals after their exposure to *S. mansoni* miracidia regardless of the genomic context of their encoding gene, with altered miRNAs originating from *MIR* gene clusters or positioned at isolated *MIR* gene loci in both intragenic and intergenic genomic contexts. (**D**) Pie chart outlining the genomic context of *MIR* genes from which the 66 miRNAs with altered abundance 16 h post the exposure of *B. glabrata* whole animals to *S. mansoni* miracidia are derived.

At 16 h post exposure of *B. glabrata* to *S. mansoni* miracidia, sRNA-Seq revealed that the abundance of 66 miRNAs was altered compared to the abundance of these miRNAs in unexposed control animals (Figure 3A). Of these 66 miRNAs with altered abundance post *S. mansoni* exposure, the degree of accumulation of eight *B. glabrata* miRNAs, including *Bgl*-miR-bantam, *Bgl*-miR-8-3p, *Bgl*-miR-9b-5p, *Bgl*-miR-29b-3p, *Bgl*-miR-100-5p, *Bgl*-miR-125-5p, *Bgl*-miR-252b-5p, and *Bgl*-miR-1985-5p, was significantly elevated at the 16 h post-exposure time point compared to their respective levels in unexposed control animals (Figure 3B). In direct contrast to this documented abundance trend, the accumulation of miRNAs *Bgl*-miR-let7-5p, *Bgl*-miR-2a-2-3p, *Bgl*-miR-216a-5p, *Bgl*-miR-252a-5p, and *Bgl*-miR-281-5p was revealed by sRNA-Seq to be significantly reduced in *B. glabrata* 16 h post exposure to *S. mansoni* miracidia compared to the respective abundance level of each of these five miRNAs in unexposed control animals (Figure 3B). Documentation of elevated and reduced miRNA abundance provided additional evidence of a biologically relevant response to *S. mansoni* miracidia exposure by *B. glabrata* and not that the observed alteration to miRNA abundance was simply due to some form of generalized “*shock response*” or “*stress response*” to exposure to this parasite.

Further evidence that altered miRNA accumulation forms part of a biologically relevant molecular response by *B. glabrata* animals to *S. mansoni* miracidia exposure was provided by our subsequent demonstration that changes to the abundance of induvial miRNA sRNAs occurred regardless of the genomic context of the DNA sequence from which the miRNA precursor transcript was originally derived. More specifically, mapping of the precursor transcript sequences of miRNAs with altered abundance post *B. glabrata* exposure to *S. mansoni* miracidia to the sequencing scaffolds that were generated previously for constructing the draft *B. glabrata* genome [12] revealed the diversity of the genomic context of the mapped sequences (Figure 3C). Figure 3C shows that the DNA coding sequences of the *Bgl*-miR-let7, *Bgl*-miR-100, and *Bgl*-miR-125 precursor transcripts formed an *MIR* gene cluster on the antisense strand of sequencing scaffold 446, a sequencing scaffold that represents an intergenic region of the *B. glabrata* genome. Similarly, the DNA sequence from which the *Bgl*-miR-72 precursor is derived was also mapped to a sequencing scaffold that represented an intergenic region of a *B. glabrata* chromosome; however, the *Bgl*-miR-72 precursor was determined to form an individual and isolated *MIR* gene locus on the sense strand of sequencing scaffold 169.

Mapping of the genomic context of the precursor transcripts of miRNAs with altered abundance 16 h post exposure of *B. glabrata* to *S. mansoni* miracidia showed that other *MIR* gene loci were also intragenic in their genomic positioning, which was derived from the introns of protein-coding *B. glabrata* genes. For example, the DNA sequences coding for the precursor transcripts of *Bgl*-miR-71, *Bgl*-miR-2a-1, *Bgl*-miR-2d, *Bgl*-miR-2b, and *Bgl*-miR-2a-2 were determined via mapping to form an *MIR* gene cluster between two exons of a *B. glabrata* protein-coding gene on the sense strand of sequencing scaffold 1244. Furthermore, it is important to note here that individual miRNAs derived from this *MIR* gene cluster displayed differences in their expressional response to *S. mansoni* miracidia exposure. Specifically for this *MIR* gene cluster, the degree of abundance change of *Bgl*-miR-71 was much greater than that determined for the other four miRNAs that also belonged to the same *MIR* gene cluster on sequencing scaffold 1244. This finding again provides further evidence that the miRNA abundance changes reported here represent an actual biologically relevant response at the molecular level by *B. glabrata* following its exposure to *S. mansoni* miracidia.

The performed mapping exercise also revealed that in contrast to this intragenic *MIR* gene cluster, the DNA sequence from which the *Bgl*-miR-190 precursor transcript is derived was determined to be positioned on its own in an intron of a *B. glabrata* protein-coding gene on the antisense strand of sequencing scaffold 42 (Figure 3C). In addition to these selected examples, mapping of the genomic positioning of the precursor transcripts of all 66 *B. glabrata* miRNAs with altered abundance 16 h post exposure to *S. mansoni* miracidia revealed that 84% (n = 55) of the precursor transcript coding sequences were positioned in an intergenic position of a *B. glabrata* chromosome (Figure 3D). Of these 84% intergenic miRNAs, 37% (n = 24) were mapped to *MIR* gene clusters on *B. glabrata* sequencing scaffolds, and 47% (n = 31) were determined to form an isolated *MIR* gene locus (Figure 3D). The remaining 16% (n = 11) of mapped precursor transcripts were determined to be intragenic, with 10% (n = 7) of these intragenic miRNAs belonging to a *MIR* gene cluster and 6% (n = 4) mapped to an isolated intron position within a *B. glabrata* protein-coding gene (Figure 3D). When taken together, the data presented in Figure 3 provide strong evidence that a miRNA-directed molecular response plays a crucial role in the immune response of *B. glabrata* to *S. mansoni* infection.

**Figure 4:**
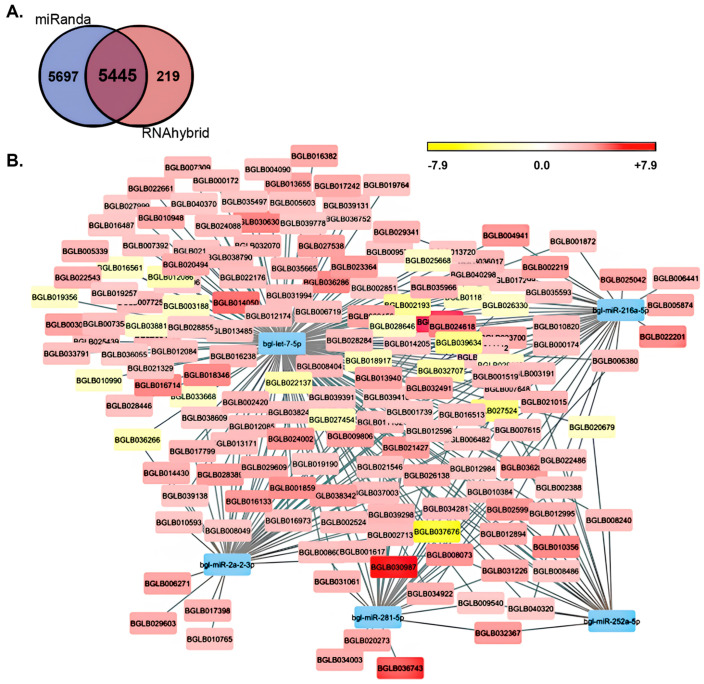
Interaction map of the putative target genes of the five *Biomphalaria glabrata* miRNAs with significantly reduced abundance 16 h post exposure to *Schistosoma mansoni* miracidia. (**A**) Venn diagram showing the number of predicted target genes for the five significantly reduced *B. glabrata* miRNAs 16 h post exposure to *S. mansoni* miracidia following target gene assessment using the miRanda and RNAhybrid prediction tools, respectively. (**B**) miRNA/target gene interaction map for the five significantly reduced *B. glabrata* miRNAs (blue blocks) post exposure to *S. mansoni* miracidia, including putative target genes both with down-regulated (yellow blocks) and up-regulated (red blocks) levels of expression.

Based on the use of miRanda and RNAhybrid algorithms for the detection of putative miRNA target sites within the 3′ UTRs of protein-coding genes, 5445 *B. glabrata* mRNAs were identified as potential targets of the 13 miRNAs that were significantly altered in their abundance: either up- (*Bgl*-miR-bantam, *Bgl*-miR-8-3p, *Bgl*-miR-9b-5p, *Bgl*-miR-29b-3p, *Bgl*-miR-100-5p, *Bgl*-miR-125-5p, *Bgl*-miR-252b-5p, and *Bgl*-miR-1985-5p) or down-regulated (*Bgl*-miR-let7-5p, *Bgl*-miR-2a-2-3p, *Bgl*-miR-216a-5p, *Bgl*-miR-252a-5p, and *Bgl*-miR-281-5p) in their abundance 16 h post exposure of *B. glabrata* to *S. mansoni* miracidia (Figure 4A). Interestingly, the vast majority of identified putative target genes (97%; n = 5285) were predicted to be targeted by the five miRNAs with reduced abundance 16 h after *B. glabrata* exposure to *S. mansoni* miracidia, including miRNAs *Bgl*-miR-let7-5p, *Bgl*-miR-2a-2-3p, *Bgl*-miR-216a-5p, *Bgl*-miR-252a-5p, and *Bgl*-miR-281-5p (Figure 4B). Via comparison of the expression level of those genes putatively identified as potential target genes of these five down-regulated miRNAs in unexposed *B. glabrata* individuals to the 16 h post-exposure time point, 303 genes were determined to have a significantly altered level of expression (Figure 4B) (Table S1).

Of these 303 genes with significantly altered expression, 158 formed putative targets of the *Bgl*-miR-let7-5p miRNA, with 137 (86.7%) genes having an elevated degree of expression and 21 (13.3%) genes revealed to have reduced transcript abundance levels in response to decreased *Bgl*-miR-let7-5p abundance (Table S1). Forty-nine putative *Bgl*-miR-2a-2-3p target genes were revealed to have significantly altered expression at the 16 h post-exposure time point. Of these, 43 (87.8%) putative targets had increased expression, and 6 (12.2%) genes had decreased expression (Table S1). For miRNA *Bgl*-miR-216a-5p, 40 putative targets were revealed to have significantly altered expression 16 h post *B. glabrata* exposure to *S. mansoni* miracidia, with 34 (85%) genes being increased in abundance and six (15%) putative targets showing a reduced level of expression. For down-regulated miRNA *Bgl*-miR-252a-5p, 20 (90.9%) of 22 putative target genes identified were determined to have significantly elevated expression, while only two (9.1%) of the identified targets were revealed to have significantly reduced expression. Thirty-three (97.1%) of the thirty-four putative target genes of *Bgl*-miR-281-5p with significantly altered expression were revealed to have increased expression, while only a single (2.9%) putative target gene of down-regulated miRNA *Bgl*-miR-281-5p was determined to be decreased in its level of expression 16 h post exposure of *B. glabrata* to *S. mansoni* miracidia (Table S1). Our demonstration that 88% (n = 267/303) of the putative target genes had a significantly elevated level of expression in response to the significantly reduced abundance of miRNAs *Bgl*-miR-let7-5p, *Bgl*-miR-2a-2-3p, *Bgl*-miR-216a-5p, *Bgl*-miR-252a-5p, and *Bgl*-miR-281-5p at the 16 h post-exposure time point formed a highly encouraging result (Figure 4B) (Table S1), a result that further highlighted the requirement of miRNA-directed gene expression regulation as part of the molecular response of *B. glabrata* to *S. mansoni* miracidia exposure.

Another highly interesting result stemming from our target gene expression analysis was that six of the putative target genes with significantly elevated expression have been previously associated with immune responses in a range of animal species [26,27,28,29,30,31] (Table 1). Of the six immune response genes identified, the *B. glabrata* locus *BGLB025042* showed the highest level of expression up-regulation, with its abundance increased by 13.4-fold (Table 1). The *BGLB025042* transcript is targeted by only one of the five down-regulated miRNAs, namely *Bgl*-miR-216a-5p, and via its analysis in the Kyoto Encyclopedia of Genes and Genomes (KEGG) database, it was assigned the KEGG orthology identifier (KO ID) K13908, an identifier that corresponds to the KEGG definition of a gene that encodes a mucin-5B protein. The *B. glabrata* transcript *BGLB027538*, which was determined to only form a putative target of miRNA *Bgl*-miR-let7-5p, was increased by 11.6-fold at the 16 h post-exposure time point. KEGG analysis assigned the KO ID K03985 to the *BGLB027538* transcript, an identifier that is defined as a gene that encodes an urokinase receptor (Table 1). Of the five significantly down-regulated miRNAs identified in this study, the transcript encoded by the *B. glabrata* locus *BGLB029609*, which showed a 9.7-fold increase in its expression level at the 16 h post-exposure time point, was revealed to form a putative regulatory target of both *Bgl*-miR-let7-5p and *Bgl*-miR-2a-2-3p. Further, analysis of this putative target transcript in the KEGG database assigned the KO ID of K06787 to the *BGLB029609* transcript, a KEGG identifier that defines the gene as one that encodes an endothelial cell adhesion molecule (Table 1).

The level of expression of the transcript encoded by *B. glabrata* locus *BGLB011132*, a transcript determined to form a putative target gene of down-regulated miRNAs *Bgl*-miR-let7-5p and *Bgl*-miR-216a-5p, was increased by 7.3-fold at the 16 h post-exposure time point compared to its level of expression in unexposed *B. glabrata* animals. KEGG analysis of the *BGLB011132* transcript assigned a KO ID of K10380 to the transcript to identify it as encoding an ankyrin protein (Table 1). At 16 h post exposure of *B. glabrata* to *S. mansoni* miracidia, the expression level of transcripts *BGLB002524* and *BGLB012596* was elevated by a similar degree, namely a 5.9- and 5.6-fold increase to transcript abundance, respectively. The *BGLB002524* transcript was determined to form a putative target of two of the five down-regulated miRNAs, including *Bgl*-miR-let7-5p and *Bgl*-miR-281-5p, and to encode for an actin β/γ 1 subunit according to the assigned KO ID K05692 following KEGG database analysis. Three of the five down-regulated miRNAs, specifically *Bgl*-miR-let7-5p, *Bgl*-miR-2a-2-3p, and *Bgl*-miR-216a-5p, were determined to putatively regulate the expression of the *BGLB012596* transcript, a transcript that, via its assessment in the KEGG database, encodes a histone deacetylase 4 protein (KO ID, K11406) (Table 1). Taken together, (1) the identification of six target genes, all with significantly elevated expression 16 h post exposure of *B. glabrata* to *S. mansoni* miracidia (Table 1); (2) the mapping of target sites within these six genes for each of the five miRNAs with significantly down-regulated abundance at the assessed time point (Figure 4); and (3) the previous demonstration of the involvement of the six identified target genes in immune responses in a range of animal species [32,33,34,35,36,37] provides further strong evidence that a miRNA-directed gene expression regulatory mechanism mediates an important role in the molecular response of *B. glabrata* following its exposure to the *S. mansoni* parasite.

## 4. Discussion

Molecular genomic approaches have proven to be a highly useful investigative tool to achieve significant advancement on our understanding of schistosomiasis. However, to date, there has been relatively little research undertaken at the intermediate host stage of the *S. mansoni* life cycle as part of schistosomiasis in humans. With the increased recognition of the importance of the central regulatory role mediated by miRNA-directed control of gene expression in both pathogen defense and host–parasite interaction, this study aimed to address this current knowledge gap via the identification of the requirement of a miRNA-directed molecular response by *B. glabrata* after its exposure to *S. mansoni* miracidia. More specifically, via analysis of the expression trends of the genes that encode key protein machinery of the miRNA pathway, including the tissue localization of the functional core of miRISC, i.e., the *Bgl*-Ago2 protein, to the site of *S. mansoni* sporocyst maturation during the chronic stage of this parasitic disease, together with profiling the abundance trends of miRNA sRNAs and their putative target genes in the acute stage of *S. mansoni* infection immediately post exposure of *B. glabrata* to *S. mansoni* miracidia, we provide strong evidence of the central requirement of a miRNA-mediated molecular response as part of this host–parasite interaction.

In mollusks, as in other animal species, the production of a mature miRNA-silencing signal commences in the nucleus of the cell. In the nucleus, a miRNA precursor transcript, termed the primary-miRNA (pri-miRNA), is initially processed. The pri-miRNA itself forms either directly from the folding of the ncRNA transcription product generated by the action of RNA polymerase II (Pol II) from a miRNA (*MIR*) gene or from the dsRNA folding structure formed from an intronic sequence post splicing of a protein-coding mRNA transcript. After this initial processing of the pri-miRNA, an intermediate precursor transcript termed the precursor miRNA (pre-miRNA) is formed, and due to the highly conserved stem-loop dsRNA folding structure displayed by all animal pre-miRNAs, the pre-miRNA intermediate is exported to the cytoplasm of the cell. In the cytoplasm, the pre-miRNA undergoes sequential rounds of processing until, ultimately, the mature miRNA silencing signal is produced [27,28,29].

Our initial homology-based searches identified *B. glabrata* homologs for each of the searched for pieces of protein machinery that perform a core functional role in either the production or action stage of the animal miRNA pathway. Analogous to its counterparts in other invertebrates [31,38], the *B. glabrata* Drosha protein was revealed to harbor two RNase III (RIBOc) domains: a functional domain that is essential for the ability of Drosha to catalyze the cleavage of pri-miRNA transcripts to produce the pre-miRNA processing intermediate [30]. In addition, *Bgl*-Drosha was determined to possess a single DSRM, a functional domain that directs (1) the binding of Drosha to its dsRNA substrates and (2) the interaction of Drosha with its functional partner protein DGCR8, an interaction that ensures accurate and efficient pre-miRNA production via Drosha-catalyzed processing of the pri-miRNA precursor transcript [30,31,39,40]. Similarly, the annotation of the functional domain landscape of the *B. glabrata* DGCR8 candidate protein strongly inferred that the correct *Bgl*-DGCR8 homolog to the *Hsa*-DGCR8 that was used as the reference protein for this search had been identified. More specifically, structurally characterized members of the DRB protein family that have been demonstrated to be essential for the production stage of the miRNA pathway include DGCR8 in *Drosophila melanogaster* [41], *trans*-activation response RNA-binding protein (TRBP) in *C. elegans* [42], and Hyponastic leaves1 (HYL1) in *Arabidopsis thaliana* [43], and all house two DSBMs in their C-terminus. In addition, the conserved functional domain organization of these three miRNA pathway-specific DRB proteins has been shown in each of these three experimental model species to (1) direct the binding of the DRB protein to its stem-loop-structured dsRNA substrates or (2) mediate its interaction with its functional partner protein, a Dicer endonuclease [41,42,43].

The domain architecture of the *B. glabrata* protein homologs of *Hsa*-Exp5 and *Hsa*-Ran identified via our homology-based search approach exhibited significant conservation with their homologous counterparts across other animal species [44,45]. Namely, *Bgl*-Exp5 was demonstrated to contain an Exportin (XPO) domain and an Importin-β N-terminal (IBN_N) domain in its N-terminal half, and the *Bgl*-Ran protein was mainly comprised of a single Ras-related nuclear (Ran) domain (Figure 1B), which is highly similar functional domain architecture to that previously reported for *Hsa*-Exp5 and *Hsa*-Ran [44,45]. Exportin-5, in coordination with Ran, facilitates the export of pre-miRNA transcripts that are bound by Exp5 in the nucleus to the cytoplasm of the cell [39]. Therefore, considering that the gene expression level of *Bgl-Drosha*, *Bgl-DGCR8*, and *Bgl-Ran* was demonstrated to be significantly elevated 16 h post exposure of *B. glabrata* to *S. mansoni* miracidia, it was somewhat unexpected to document that *Bgl-Exp5* transcript abundance was reduced and not elevated to a similar degree as the other core protein machinery of the *B. glabrata* miRNA pathway (Figure 1C). However, a downward expression trend was previously reported for *Bgl-Exp5* in *B. glabrata* whole animals 12 h after their exposure to miracidia of the *S. mansoni* parasite [46]. At 42 h post exposure, *Bgl-Exp5* expression returned close to its levels in unexposed *B. glabrata* animals (Figure 1C), and similarly, previous research [46] showed *Bgl-Exp5* transcript abundance to be elevated well above its normal level of expression in unexposed animals 7 days post *B. glabrata* exposure to *S. mansoni* miracidia. Considered together, these expression trends indicate that *Bgl-Exp5* levels decrease rapidly post initial exposure; however, *Bgl-Exp5* transcript abundance appears to return to its unexposed levels or to even higher levels following the very early stages of *S. mansoni* miracidia exposure.

Once in the cytoplasmic compartment and after release by the Exp5/Ran exportation complex, the PAZ domain, together with its C-terminal-localized DSRM domain, facilitates the recognition and binding of the stem-loop-structured dsRNA of the exported pre-miRNA by Dcr [40]. The C-terminus-positioned RIBOc domains of Dcr then catalyze the next stage of processing of the miRNA precursor transcript: cleavage events that liberate the miRNA/miRNA* duplex from the stem arms and loop region of the pre-miRNA transcript [30,47]. Annotation of each of these essential functional domains in the candidate protein identified in this study, together with the conserved positioning of each identified domain, strongly inferred that the homology search approach adopted here identified the correct *Bgl*-Dcr homolog (Figure 1). After processing by Dcr [30], mature miRNA/miRNA* duplexes are subsequently loaded by Ago2, which forms the functional core of miRISC [23,48]. As part of miRISC, Ago2 is involved in duplex strand separation, a process that results in the degradation of the miRNA* passenger strand and retainment of the now fully mature miRNA guide strand by miRISC [23,48]. Annotation of the Ago protein family-specific N terminal domain, DUF1785 domain, a L2 linker region, and MID and PIWI domains [24,49] in addition to the PAZ domain all together strongly inferred that our homology search had indeed identified the correct *B. glabrata* Ago2 candidate protein (Figure 1B). Further support that the correct *Bgl*-Ago2 protein candidate had been identified was supplied by our finding that only a single suitable *B. glabrata* candidate could be identified with any degree of reliability via our homology-based search approach. In addition, a single Ago2 protein forming the functional core of miRISC appears to form a conserved feature of the miRNA pathway of other molluskan species [28,29]. miRISC uses the Ago2-loaded miRNA as a guide to identify regulatory transcripts that house target sequences harbored in the 3′ UTR of the target transcript to direct expression regulation of the protein-coding gene [50]. Therefore, the identification of PAZ (recognition and binding of siRNA and miRNA 3′ terminal nucleotides), MID (binding of the 5′ terminal nucleotides of the loaded sRNA), and PIWI (facilitates Ago interaction with ssRNA target transcripts of the loaded sRNA) domains and confirmation of their conserved positions within the identified candidate protein [24,49] allow for confident prediction that the identified *Bgl*-Ago2 protein performs a similar core functional role central to the action stage of the *B. glabrata* miRNA pathway.

As shown for *Bgl-Drosha*, *Bgl*-*DGCR8,* and *Bgl*-*Ran* expression, the expression of *Bgl*-*Dcr* and *Bgl*-*Ago2* was elevated to the most significant degree in *B. glabrata* whole animals 16 h post exposure to *S. mansoni* miracidia (Figure 1C). In contrast to this elevated gene expression trend at the 16 h post-exposure time point, the expression of the encoding loci of these five pieces of core protein machinery had either started to or had fully returned to their respective unexposed (control) level at the 42 h post-exposure time point (Figure 1C). In addition, although *Bgl*-*Exp5* expression remained below its level of expression documented in unexposed control animals at the 42 h time point, *Bgl-Exp5* expression had also started to return to its unexposed level at the second assessed short-term time point. The differential regulation of *Bgl-Drosha*, *Bgl-DGCR8*, *Bgl-Exp5*, *Bgl-Ran*, *Bgl-Dcr,* and *Bgl-Ago2* expression documented here for the 16 h short-term time point compared to the 42 h short-term time point in *B. glabrata* following its exposure to *S. mansoni* miracidia (Figure 1C) highlights the intricate interplay between the miRNA pathway of the host snail and the invading parasite during the very early stages of exposure to this parasite. More specifically, the documented expression changes to the six analyzed pieces of core protein machinery of the *B. glabrata* miRNA pathway, providing an elegant demonstration of the dynamic adjustments required in miRNA-mediated target gene expression regulation pathways in the host, which may influence host immune responses, tissue repair processes, or other molecular-based defense mechanisms to appropriately respond to the invading *S. mansoni* parasite. Furthermore, a greater degree of expression response at the 16 h post-exposure time point compared to the 42 h time point identifies this very early-stage time point for the focus of future investigations into the specific miRNAs and their respective target genes to attempt to identify the most pivotal miRNA/target gene expression modules to target for the development of intervention strategies against schistosomiasis in humans.

Considering that Ago2 has been demonstrated to form the functional core of miRISC [23] via its use of bound miRNA sRNAs as guides to direct RNA silencing of target genes primarily via a translational inhibition mechanism of RNA silencing [50,51], *Bgl*-Ago2 was selected for further characterization. The initial detection of *Bgl*-Ago2 protein abundance (Figure 2A) confirmed the transcriptomic analysis (Figure 1C); that is, Ago2 abundance was elevated in *B. glabrata* whole animals at 16 and 42 h post exposure to *S. mansoni* miracidia. Following this finding and our demonstration that the Ago2 antibody generated for use in this study was specific to the *Bgl*-Ago2 protein via our inability to detect hybridization products in the *S. mansoni* miracidia sample (Figure 2A), immunolocalization of *Bgl*-Ago2 clearly showed the presence of *Bgl*-Ago2 in the digestive system of chronically infected *B. glabrata* animals, specifically localizing to the region of the tract where *S. mansoni* sporocysts formed 20 days post exposure of *B. glabrata* to *S. mansoni* miracidia (Figure 2). Under normal physiological conditions, Ago2 and thereby miRISC are predominantly localized to the cell cytoplasm [52], where Ago2-catalyzed miRISC directs miRNA target gene expression regulation at the post-transcriptional level. Moreover, according to the protein localization data presented in Figure 2, upon parasitic infection, Ago2 may undergo spatial and temporal changes in its localization as part of the activation of the host immune response to parasite invasion. Such changes in *Bgl*-Ago2 tissue localization could be driven by various factors, including the induction of immune signaling pathways, the release of pro-inflammatory cytokines, or the activation of cellular stress responses [53,54]. For example, Ago2-mediated, miRNA-directed RNA silencing has been shown to play a crucial role in host defense against parasitic infection by regulating the expression of immune-related genes and modulating host–pathogen interactions [44]. In *B. glabrata*, Ago2-catalyzed miRISC may contribute to the clearance of parasites by targeting parasite-derived transcripts or modulating the expression of host genes involved in immune responses. Additionally, Ago2-dependent mechanisms such as miRNA-directed translational repression of target gene expression may influence the replication and dissemination of parasites within the host in an attempt by the host to negate the negative impact of parasite infection. Further, the *Bgl*-Ago2 protein tissue localization findings (Figure 2), when taken together with the demonstration of significant altered abundance of numerous *B. glabrata* miRNAs (Figure 3), suggests that following exposure and infection by the *S. mansoni* parasite, *B. glabrata* may potentially secrete an altered miRNA profile in a defense responsive to the parasite. Nonetheless, the analysis in Figure 2 does identify *B. glabrata* Ago2 as a protein that performs an essential role as part of the molecular response of the snail to the *S. mansoni* parasite. Therefore, future strategies that use a molecular approach to enhance Ago2 activity or that restore miRNA-mediated regulation of immune responses may greatly bolster the host defense mechanisms of *B. glabrata* to limit the persistence of the *S. mansoni* parasite. Conversely, the use of a molecular approach to inhibit the Ago2-dependent pathways in the parasite itself could disrupt the ability of the parasite to evade the immune surveillance system of the host or impair the survival and transmission of the parasite. Further research is therefore needed in the future to elucidate the specific roles played by the Ago2 protein in the *B. glabrata* host–parasite interactions with *S. mansoni* to fully evaluate the use of *Bgl*-Ago2 and thereby *Bgl*-Ago2-mediated systems as potential antiparasitic therapeutic targets.

To further uncover the degree to which miRNA-mediated molecular mechanisms are involved in regulating *B. glabrata* host–*S. mansoni* parasite interactions, the miRNA landscape of *B. glabrata* 16 h post exposure to *S. mansoni* miracidia was profiled for comparison to that of control (unexposed) animals. Our profiling focused on previously reported miRNAs, and of the 66 known miRNAs identified in the control and exposed whole-animal samples, 13 miRNAs were determined to have significantly altered expression. Specifically, miRNAs *Bgl*-miR-bantam, *Bgl*-miR-8-3p, *Bgl*-miR-9b-5p, *Bgl*-miR-29b-3p, *Bgl*-miR-100-5p, *Bgl*-miR-125-5p, *Bgl*-miR-252b-5p, and *Bgl*-miR-1985-5p were significantly elevated in their abundance, while five miRNAs, including *Bgl*-miR-let7-5p, *Bgl*-miR-2a-2-3p, *Bgl*-miR-216a-5p, *Bgl*-miR-252a-5p, and *Bgl*-miR-281-5p, were significantly reduced in abundance (Figure 3). Interestingly, of the eight significantly up-regulated miRNAs, miR-bantam-3p, miR-8-3p, and miR-1985-5b, were previously shown to be up-regulated in their abundance in oyster (*Crassostrea gigas*) hemocytes following bacterial challenge [45]. Also in oyster hemocytes, miR-100 was revealed to have enhanced abundance under heat-stress challenge [45]. In *B. glabrata*, the enhanced abundance of these miRNAs is likely the result of either the transcriptional induction of the loci that encode the precursor transcripts of these miRNAs or could be the result of an altered status of their biogenesis [48] or degradation [23], triggered as part of the immune response of *B. glabrata* upon its exposure to *S. mansoni* miracidia. Furthermore, in addition to oysters, the immune response regulation of these miRNAs was also previously reported in other animal species [50]. For example, miR-100-5p was observed to promote the anti-virus immune response of the shrimp species *Marsupenaeus japonicus* (Kuruma shrimp) via regulating apoptosis and phagocytosis activity [24]. Here, our analysis of miRNA abundance changes in response to *S. mansoni* miracidia was derived from whole-animal samples; however, hemocytes of the hemolymph are the likely vehicles for carrying immune response-related miRNAs to the site of infection to mediate a miRNA-directed immune response, as was demonstrated previously in oysters [8]. Therefore, our future studies should specifically concentrate on the profiling of the miRNA payload of *B. glabrata* hemocytes immediately (12 to 16 h) after its exposure to *S. mansoni* miracidia to further uncover the full degree of the miRNA-directed immune response of *B. glabrata* to this parasite.

It was somewhat unexpected to find that the vast majority (~97%, n = 5285) of the 5445 putative target genes identified for the 13 *B. glabrata* miRNAs with significantly altered abundance formed potential regulatory targets of the five significantly down-regulated miRNAs. This finding suggested that a particularly complex regulatory landscape is orchestrated by the down-regulated miRNAs *Bgl*-miR-let7-5p, *Bgl*-miR-2a-2-3p, *Bgl*-miR-216a-5p, *Bgl*-miR-252a-5p, and *Bgl*-miR-281-5p in *B. glabrata* post *S. mansoni* exposure. Among the putative targets identified for the five significantly down-regulated miRNAs were six genes that encode proteins previously associated with immune responses [32,33,34,35,36,37]. More specifically, and based on KEGG definitions, immune response genes that encode protein homologs of mucin-5B, urokinase receptor, endothelial cell adhesion molecule, ankyrin, actin β/γ 1, and histone deacetylase 4 were determined to form putative regulatory targets of one or more of the five significantly down-regulated miRNA in *B. glabrata* after its exposure to *S. mansoni* miracidia (Table 1). In humans and in mice (*Mus musculus*), the mucin-5B protein is encoded by one of five gel-forming mucin genes [55], with all five gene family members encoding for high-molecular-weight, heavily glycosylated proteins (glycoconjugates) produced by epithelial tissues [56]. In humans, the mucin-5B protein is primarily found in whole saliva and in normal lung and cervical mucus, with *Hsa*-mucin-5B abundance demonstrated to be altered in respiratory conditions such as chronic rhinosinusitis (chronic sinusitis) and chronic obstructive pulmonary disease (COPD) and in gastric diseases that result from *Helicobacter pylori* infection [56,57]. Furthermore, elevated *Hsa*-mucin-5B levels in each of these conditions indicates that altered mucin-5B abundance may play a role in the pathogenesis of these conditions. Most snail species produce mucins as part of their mucous secretions, with such secretions performing a wide range of biological functions, including lubrication, adhesion, and microbial protection [58]. The foot of a snail has the most contact with surfaces that are potentially contaminated with pathogens and parasites, with mucus secretion along the length of the foot structure likely protecting the snail against the pathogenic microorganisms that it encounters [34]. Moreover, whole mucus and/or mucosal extracts from the foot mucus of the giant African land snail (*Achatina fulica*) showed promising antibacterial activity against the Gram-positive bacterial species *Bacillus subtilis* and *Staphylococcus aureus* [59], and similarly, the foot mucus of the brown garden snail (*Helix aspersa*) possesses antimicrobial activity against numerous strains of *Pseudomonas aeruginosa* [60]. When these previous findings are considered together with our demonstration that the expression of a *B. glabrata* gene defined as encoding a protein product with homology to mucin-5B is significantly elevated in response to significantly reduced abundance of the putative regulatory miRNA *Bgl*-miR-216a-5p, it is tempting to speculate that the *Bgl*-miR-216a-5p/*Bgl*-*mucin-5B* regulatory module may play an analogous role in this host–parasite interaction. More specifically, elevated mucin-5B production and therefore increased foot mucus secretion may offer an enhanced degree of protection to *B. glabrata* against parasite infection post exposure to *S. mansoni*.

Following the 13.4-fold reduced expression of the mucin-5B encoding transcript *BGLB025042*, transcript *BGLB027538,* which via KEGG analysis was defined as encoding an urokinase receptor homolog, was reduced in its level of expression by 11.6-fold (Table 1). The expression of the urokinase receptor (uPAR) was revealed to be localized to leukocytes, including monocytes and macrophages, as well as in granulocytes and immature dendritic cells, with uPAR shown to be involved in mediating the migration of mobile immune cell types across the blood barrier and into neighboring tissues [61]. Mediating the movement of mobile cells of the immune system out of the circulation and into tissues identifies a role for uPAR in controlling crucial early events in inflammation, immune responses against pathogen infection, and in tissue remodeling following injury [62]. Moreover, uPAR was also demonstrated to be able to regulate cell adhesion, migration, and proliferation as well as to protect cells from becoming targets of apoptosis and anoikys [49]. It is important to note here that post infection by the *S. mansoni* parasite, *B. glabrata* digestive gland epithelium is poorly preserved, with these cells displaying varying degrees of atrophy [63]. Therefore, increased expression of the *Bgl-uPAR* homolog (Table 1) in response to decreased abundance of its putatively regulating miRNA, i.e., *Bgl*-miR-let7-5p (Figure 3), tentatively suggests that the *Bgl*-miRlet7-5p/*Bgl-uPAR* expression module may be altered in *B. glabrata* to combat the tissue damage caused by *S. mansoni* miracidia infection.

KEGG analysis of transcript *BGLB029609*, a putative target of miRNA-directed expression regulation by two of the five down-regulated miRNAs, namely *Bgl*-miR-let7-5p and *Bgl*-miR-2a-2-3p, with a 9.7-fold increase in its abundance post initial parasite exposure, defined the transcript as encoding an endothelial cell adhesion molecule (Table 1). Endothelial cell adhesion molecules (ECAMs) have been shown to play key roles in several pathologies, including inflammatory disease [25,32]. In addition, ECAMs belong to a family of immunoglobulin-like molecules that are expressed on the surface of endothelial cells and that have been demonstrated to engage with leukocyte counter-receptors to mediate firm adhesion and/or *trans*-endothelial migration [36]. For example, the ECAMs that belong to the selectin, integrin, and immunoglobulin gene superfamily of adhesion receptors mediate different steps of leukocyte migration from the circulatory system to sites of inflammatory foci in animal tissues [25]. In addition, it was reported that in human protozoan parasitic diseases, the vascular endothelium, a monolayer of endothelial cells, also directs a regulatory role in inflammation and immune cell trafficking, with endothelial cells serving as a replicative niche for many bacterial, viral, and protozoan infectious diseases [33]. Although, to date, the functional roles directed by ECAMs have not been studied to any great detail in mollusk‒parasite interactions, based on the findings reported here, it can be suggested that molecular alteration of either the *Bgl*-miR-let7-5p/*Bgl-ECAM* and/or *Bgl*-miR-2a-2-3p/*ECAM* expression module is initiated by *B. glabrata* as part of its early immune responses to infection by the *S. mansoni* parasite.

As the putative regulatory target of down-regulated miRNAs *Bgl*-miR-let7-5p and *Bgl*-miR-216a-5p, *B. glabrata* transcript *BGLB011132* was increased in its abundance by 7.3-fold 16 h post parasite exposure (Table 1). Initial BLAST searching using the *BGLB011132* transcript revealed the transcript to have a high degree of similarity to previously characterized *Ankyrin repeat domain-containing protein 17-like* genes (*Ankrd17*), a finding that was confirmed via KEGG analysis, which defined *BGLB011132* as a transcription product of an Ankyrin (ANK) encoding gene (Table 1). Interestingly, previous research demonstrated in vitro that a miRNA sRNA-silencing signal derived from bovine leukemia virus (BLV) targets a bovine *Ankrd17* gene for expression regulation, a finding that readily identifies an important role for this retrovirus-derived miRNA in the pathogenicity of BLV [64]. In another in vitro study using HeLa cells, Ankrd17 was shown to contribute to the inflammatory response of this cell line to infection by the Gram-negative bacteria *Shigella flexneri* via a Nucleotide binding oligomerization domain containing 2 (Nod2) intracellular receptor-initiated response pathway [33]. Members of the ANK protein family were shown to mediate the attachment of integral membrane proteins to the cytoskeleton of cells [65], and in mice, up-regulation of *ANK* gene expression was shown to increase the susceptibility of exposed mice to rodent malaria infection, specifically the merozoite stage of the life cycle of the parasite [66]. Considering these previous research findings, it is possible that down-regulated *Bgl*-miR-let7-5p and *Bgl*-miR216a-5p abundance (Figure 3) in combination with elevated expression of their putative target gene, *Bgl-ANK* (Table 1), is required by *B. glabrata* to attempt to mount an effective immune response to *S. mansoni* infection.

Histone deacetylase 4 (HDAC4) is a class IIa deacetylase and, like all other HDACs, is an epigenetic enzyme responsible for the removal of acetyl groups from specific lysine residues in the amino terminal tails of histone proteins [67]. Histone proteins form nucleosomes as part of chromatin, with acetyl group removal from the lysine residues of histone amino terminal tails associated with heterochromatin formation and thereby termination of active gene expression [68]. HDAC4 has been previously associated with general immune system responses and with specific antipathogen responses, including the silencing of retrovirus sequences [69] and the repression of the manipulation of host cellular machinery by influenza A virus (IAV) for its replication: an HDAC4-functional role documented as part of the innate immune response in humans to IAV [32]. More specifically, HDAC4 appears to specifically deacetylate loci to suppress the expression of targeted loci that encode proteins that normally negatively regulate the activity of pro-inflammatory and antiviral cytokines against IAV infection via the Toll-like receptor 3 (TLR3) innate immune signaling pathway [70]. A similar anti-viral immune response role to foot-and-mouth disease virus (FMDV; coxsackievirus A16) was assigned to HDAC4 in a number of mammalian species as part of an interferon-mediated response in these host species to infection by FMDV [71]. Therefore, by inference, our demonstration here that *Bgl-HDAC4* expression is significantly elevated (Table 1) due to the reduced abundance of its putatively regulating miRNAs *Bgl*-miR-let7-5p, *Bgl*-miR-2a-2-3p, and *Bgl*-miR-216a-5p (Figure 3) could conceivably be functioning in an analogous manner in *B. glabrata* whole animals in response to their exposure to the *S. mansoni* parasite. Moreover, elevated *Bgl*-HDAC4 protein activity is required to epigenetically suppress the activity of repressors of *B. glabrata* TLR3 signaling pathways to ensure that pro-inflammatory and pro-immune responses are activated as part of the initial stages of the defense response of *B. glabrata* to infection by *S. mansoni* miracidia.

Of the six different isoforms of actin documented in humans, only the beta- (β-) and gamma- (γ-) actin isoforms can be found in each cell type [72]. Moreover, although β- and γ-actin are almost identical at the amino acid sequence level, these two actin isoforms are encoded by different genes [73]. However, due to their amino acid sequence similarity, KEGG analysis of the *B. glabrata* transcript *BGLB002524* was unable to distinguish between the β- and γ-actin isoforms. Nonetheless, the *BGLB002524* transcript was identified as a putative target of miRNA-directed gene expression regulation of both *Bgl*-miR-let7-5p and *Bgl*-miR-281-5p, and furthermore, in response to significantly reduced abundance of both *Bgl*-miR-let7-5p and *Bgl*-miR-281-5p (Figure 3), the expression level of *BGLB002524* was significantly elevated (Table 1). Both β- and γ-actin play roles in intracellular motility such as the transport of cellular organelles [74], and furthermore, it was shown that certain defects in the *ACTB* gene, which the encodes β-actin isoform, can impair neutrophil locomotion without affecting the synthesis of β-actin itself or impacting on gross neutrophil cytoskeleton morphology [75]. Therefore, a change in β-actin/γ-actin composition and/or ratio is likely to play a role in regulating the pathogenic ability of *S. mansoni* miracidia to infect *B. glabrata* whole animals. However, it is important to note here that while the use of computational prediction algorithms provide valuable hypotheses about the biological relevance of individual miRNA/target gene expression modules, experimental validation of each specific module, notably under each different cellular condition, is essential not only to confirm the functional importance of each module but also for the further advancement of our current understanding of the regulatory complexity of the biological system under study.

## 5. Conclusions

This study demonstrates the central importance of the miRNA pathway and miRNA-directed gene expression regulation as part of the molecular response of *B. glabrata* to *S. mansoni* miracidia exposure. We observed changes in core protein machinery that function at either the production or action stage of the miRNA pathway and also provide the first insights into Ago2 protein localization in tissues where sporocysts formed in long-term infected snails. We identified specific *B. glabrata* miRNAs and target genes with altered abundance post parasite exposure, particularly immune response genes. These findings highlight the crucial role of the miRNA pathway in this host–parasite interaction, which could form potential new strategies and/or avenues for controlling schistosomiasis in humans in the future.

## Figures and Tables

**Table 1:** The six *Biomphalaria glabrata* immune response genes with significantly up-regulated expression and that form putative target genes of the five miRNAs with significantly reduced abundance 16 h post exposure to *Schistosoma mansoni* miracidia.

***B. glabrata*** **Gene ID**	**Expression Fold Change**	**Targeting miRNA(s)**	**KEGG Identifier (KO ID)**	**KEGG Definition**
*BGLB025042*	+13.4	*Bgl*-miR-216a-5p	K13908	mucin-5B
*BGLB027538*	+11.6	*Bgl*-let-7-5p	K03985	urokinase receptor
*BGLB029609*	+9.7	*Bgl*-let-7-5p *Bgl*-miR-2a-2-3p	K06787	endothelial cell adhesion molecule
*BGLB011132*	+7.3	*Bgl*-let-7-5p *Bgl*-miR-216a-5p	K10380	ankyrin repeat- domain protein
*BGLB002524*	+5.9	*Bgl*-let-7-5p *Bgl*-miR-281-5p	K05692	actin β/γ 1 subunit
*BGLB012596*	+5.6	*Bgl*-let-7-5p *Bgl*-miR-216a-5p *Bgl*-miR-2a-2-3p	K11406	histone deacetylase 4

## Data Availability

Transcriptome data can be accessed online using the Gene Expression Omnibus (GEO) Database accession numbers: SRR18863902, SRR28266338, and SRR28266337. Small RNA transcriptome data can be accessed online using the GEO database accession numbers: SRR28254504, SRR28254505, and SRR28254503. Any other data can be requested via contacting the senior corresponding author of the study, S.F.C. (scummins@usc.edu.au).

## Supplementary Materials

The following supporting information can be downloaded at: https://www.mdpi.com/article/10.3390/genes15081023/s1, Figure S1: Amino acid sequences encoded by the candidate genes identified via a standard BLAST search analysis approach of core pieces of protein machinery of the *Biomphalaria glabrata* miRNA pathway; Figure S2: Microscopic analysis of sectioned *Biomphalaria glabrata* whole animals 16 h and 20 days post exposure to the miracidia of the *Schistosoma mansoni* parasite; Figure S3: Immunofluorescence assessment of Ago2 protein localization in *Biomphalaria glabrata* animals 16 h and 20 days post exposure to *Schistosoma mansoni* miracidia; Table S1: Differentially expressed putative target genes of significantly reduced *Biomphalaria glabrata* microRNAs.
