# Supplementary material for: ARGONAUTE2 Localizes to Sites of Sporocysts in the Schistosome-Infected Snail, Biomphalaria glabrata
Source: Genes (Basel). 2024 Aug 3;15(8):1023. doi: 10.3390/genes15081023 (PMC11353429; doi:10.3390/genes15081023)
Supplement: Supplementary file 1 [file genes-15-01023-s001.zip › genes-3096326-supplementary.pdf]

Figure S1A.

Bgl-Drosha (*BGLB003167*)

MNRNQSFQTSRYTFPHQQQQQQPQTIINNLMLQQQQQFRTSNQFNSERILGHQSVQNGLRNPLPFNSQKHVRPWHQNPQQSQFNQFTNPVHSNNPF
IRAASNNAECFPTMLSSPTPTNFNSNGVLTQTFPSHSVLGNNPCHFIPTHQPPPSMNVQTSVPFGSCNKFSSGQSNPDFQAPMQHYTAHKHNFASKL
NSGYEKVRTNNSRMTFPSQKYDNNKVPADLSPRYNSGSKHESSHRIGTYQLSHGQKKHDEVAVELEDNLNNSVKNKVSKSGYTTLGFNSSSKNDDRK
SQYREKRDEISRSRDEKKHISVRKHIDHADSSKYNPRKESYRETEKLNLKDGRRNSYTYRNDRNQDREKNTFSSRNERKTVSSRSKSSRFSRSPIRE
RKLRTHSSSSSSQSCKVSKRSRSPSLTYEQDLGTIHKHLSQYRQDLLCSEQLDNNDMDKLSPSKKRKIESDLAENLESEPVGYFTRSCPADLYFFKN
PETGNMEATSRMVELENRFEEDIVKRSEKRTPNIEELLPPCSTHHHHHHCSGHKSSSCDSSSSEDDDDDDDDEGAVSQIMEEWDRRKKFPNSLHPEL
WFNIKGQANDGPLCRCSIKSRQSGIRHDIYPGEMPLELCDEESNNIHRLYHYRLTMSPYTNFLTKVPTLINHDNHEYIFEGFSMFSHVKLENIPACK
LLRFNIEYTIHFIPEAAPENFTIRSLNLFNRFIFTEILDLFDMDWTGQKGGCNRFHFMPRFCRTLPDSGCKEVLSMNTVLNFLLKSAIPLVDESRVN
EIMESGESEWHTMVDNLRGMIVTNPGMKPSSIRIDQLDRKVLTLQGNPLKQNLDNDSEKEKKNTEKHNESQILEEDGSLKYPLIIHQGYRPAQLSYA
GDPVYRKAWKQYVKFRHLLNSKPKILAADKMKLKEQEEKLREIRMKKTMKREVTIELSSKGFLCTGLRSDLCQHALMIPVLMSHLRFHICLDVLENS
IDYKFVDRNLLQLSLTHTSYRTNYGTNPDHTRNSLTNCGMRQVEYGDRRIHYQNTRKRGICILVDIMSRLGKQEETVSEIPHNERLEFLGDAVIEFL
TSVHLFYMFPWLEEGGLTTYRMALVQNQHLAVLAKKLKLQDFMLYVHGPDLCHESDLQHAMANCCEAIMGALFLDGGIDVVDRIFSKALFDDDEILL
HTWNDLPKHVLQQQEPGGDRRWVKSSPILQKLEKFEESIGIRFNHIRLLAKAFTHRNIGVNPLTMGHNQRLEFLGDTVLQLVASEYLYKHYPEHHEG
HLSLLRSSLVNSRTQGLVYDDLGMSEYVIYNENAISDGVDMKTKQRADILESFVGALFVDKDLDYCTAFCNVCFFPRLKDFIMNQDWNDPKSQLQQC
CLTLREVGGGEPDIPVYKVIESIGPTNTRKYVVAVYFRGKRLARGTGHSIQQAEMAAATQALKKRAELFPILQHQRRFLERMKKGERPSDDEMANDR
RKMNKYKQKQRDKK

Figure S1B.

Bgl-DGCR8 (*BGLB021310*)

MSDCQTDITQNQRVDNQFNVSQDLGAYNDNSMEDISLSDCMEEGDNESNHGDDGSNHDGDYGDPGEEHEFEIIDQLESEEDDGIGTERSGNDRQRKE
DQDDYSSSSDDLDDTELHAMLEKGIDKDSIKKPDENQDGRPIIKHKIVLNELESDPFDILPEGWVWVTHNCGMPVYLHKETRVCTMARPYSLGSASA
RTHDIPISAIPCLQYRRQMEKLKPESQVDDCKNDSSNIEVKNSFPTEPVKTNSAVSIEVDNSKESLKQPSVNNEKKSPGKLWVVAEESSEFKTLDSQ
YGALHTGVGDYKTYQHPELNTSEAPSSLVCENIAVRLDRSCLDDSENINAHALGETGFEASISSASENLSVEVNAQNLGSQQNLNADPEEKEKHLIG
SCAINAKHGQCSEKPLICPMSWPSVGENSPSTNCLISQKNLHGDDTNASKVVTEDNLKSTVAVVAEEATSNTERSELVTSSTNPLASIKRKIESLKN
KRGGKHRKLDNGSATGWPTDNNIAVSARSSGREDTFNGAPIPNADEKLKAHAAEVKIKSAEERAKESLLDSSTVHDYCTKLFQFKTLEVKKYRSWRE
RRKHLGERSRRNRPELPPSTKLITCPIPGNMKSEGTGDNAKKREFILNPTGKSYLCILHEYMQRTLKIQPLYVFKELENSKTPYGATVMINNIEYGT
GYASSKKVAKQEAAKETLKVLIPDLFKKITDQEIKRNISDLSFFDDVKVTDPRVNELGNKVGQPSPFQLLLECLRRNFGMGNTQCEVSTKALKNQKC
EFTINVGKHSATVVAKNKREGKQLAAQAILAKLHPHVPSWGSLLRLYGTSVEKAIQKPEEMNDVKSHAPNHSVLESLREEMKKLHRQKEAIQSKGKM
IISSKDLPSKMSGVDL

Figure S1C.

Bgl-Exp5 (*BGLB016950*)

MAFTSVHLEVIHSLINAVEKVMNPLATNEERQIAHKICEEFKETSKQSFQYGLHLAEKNNSSFVRHFGLQVVEHFIKYRWQEASPADKSDLKINVFK
LVDQGTKDILEEEQHIKDGVSRILVELMKREWPQLWENLFTDFTVLCRNGETQTELVLLTLLRLTEDVVRFQNLPHARRRELLQALTSGMSSISSFF
MFTLNAHLEKYKSKTGIEAEKSCKICLCVLDTLTAFVDWINITHIIEANLLPRLSSLLLDKNLCLRASECLLLIVGRKGKISERKPIMVLFSQEAMT
VLLQAANNATEHITESTNYIFLKRLCEILLEIGKQLCYLWGSSEDTGQPPNFEMYLKALLAFTQHPSQSLRQMIYTMWLIFLRHPIASKDPVFQSVL
PMLIQCGTVCLHKVGFPSHSNSVSCDYSRLEFDTDEEFNAVLSCLRVSVVESIRTLTLMVPKLTFSVASAWLTELLNKPIDIGTGADADRGICNLSS
PSFISWDACSVFLEAVMSKLFVGDGDKSFVQEGIDLLHKALAYQMQDPLILSAVLSCLSGLFPFLNYTPQTLPQVLEKIFGAVVFNLPGQTKSTRSQ
AVKNVRVHACSVLVKICKNYPGLLLPEFRHLYDSVKQLDSDREQLSQMEKIILIEALIIVSNQFHDFARQAAFIEEVIAPVKELWSSEDFNKAFSSP
EYFMDYVGLNKAAVEPSSADTCGINRSHITYCINTILAVIKRSQWPEDYNVAQRGGFIIGGEGCSVLRNPATPYISPLLNNLMTLLKTTCSLFKPEY
LHLRHIDFVRAYDLTEHDKLNILGIPPVSVDNSDSLVYRHPLERMQIFISTVFDYGFHILGNASQCLGAEFYCVPGLSKVIIENLIVNFKLLPDFRA
KTFIRNFIKPFIQCCPKGQCSTVVLPVLYILTSDILQRLSERWLIINKRVEEEAQSEEQSDPESQEILEEHVVRQLTREYLELLVLVLTGKSVNSDV
KEEMAMEDGDVGKQTGSNPPFKELSELGVMVVGTKELFPSIIMCIINGFSWADTTVCHRCTQMLWPVVKQIVANNDMSEEAASHIFVAILTGLQLHG
EHESTQSCLLTLSFFYYETLRQKFPSLTQVLHKIPDVNVNLIKNLEVELSSRTGQEKKKKIASRRLFPALLGEVSLIGSGGLCSTAICRSCFFQPGD
QRQFSWMKWSLKIWDYHDSALRSVLLIRA

Figure S1D.

Bgl-Ran (*BGLB011892*)

MAQMDDIPTFKLVLVGDGGVGKTTFVKRHKTGEFEKKYVATLGVEVHPLVFHTSRGPIKFNVWDTAGQEKFGGLRDGYYIQGQCAIIMFDVTSRVTY
KNVPNWHRDLVRVCENIPIVLCGNKVDIKDRKVKAKTIVFHRKKNLQYYDISAKSNYNFEKPFLWLARKLVGDPNLEFVEMPALIPPEVQMDAALAR
KYEDELRIQRMQQMLAKMQEQLKASSPQQQHVNGLGKLKLKRDSAANFEALSDTEKLLEQKDAEENQQLSRAGVLNPNPNSRDIKMV

Figure S1E.

Bgl-Dcr (*BGLB002125*)

MAHSNTTGPRKGSRYHRLDDIPTDTFTPRTYQIQLLDSALKRNSLLCLGCSESKFFLALMLAKELSSVTRLSLGAGGKRTFFLTDSEEDVDLLCQTL
PNHTDLFINSCPMGLETENEKSFNDWRSLMQSNNILVCLGTKFLYAIQRNYIQLKEVNLIIFDNCHKAVEVENHPYAAIVQGIKKLTKSDRPHILGV
TAAIAGTDCNDPEKLTRTISSMEETMFAYAETSMLILSERFGCRPKESIIRCGDSELYDDHSSLKDILEDILEQCYFFFSDCRISIDDAVDNRNPCD
TPLQVLNQCLNILNILGSWCTASVAEYFIMQLDKIIKFEKNDIHKQFLRCVITTLRYLVKTFEMNFHPNYDVNELLEYTTPKVRELVTVLRKYKPEM
DFIIVSNSDSYNDDDEFSDISDDDDDSIMSGSDDEDSSGEGTKSKPMHIAVKRTADGGHERIMTFSEEEEKNLCGIVFVESRYIAFGLNKIIEEVCS
WDENLCFVKSCHITGQGLRGANGKQKVNKTYKRQEEALRKFRTQECNLVIATLELEEGIDIPKCNLVVRFDPPKDFRAYALSKGRARARDAVYVILL
DNDNFEGFNSSLKVFKGIEQVLLGENRSLSLTESLSSGEEADCEAEDDDEEMSESKDQQLTPYYTMPNTPNSPHVTMQSSIALINRYCAKLPSDAFT
HLTPHCRIESVAGEPPLYIAYLRLPINSPIKEELQGPAMKTRKLAKMAVALKMCEILHKKGELDENLVPVGKEMFLYEEEEECQNEEDMTGQARPGT
TKRNNFILKRCAEVLLNCRPLPGEPSFLYLINLKLTGPITDEQNTRGRVIYAPEDTVQTLGILLSKKIPLMPYFPVYTRSGEVTVSIDLIQDIICSE
EEILRLSKFHHFVFSGVLHLEKDPMDFDYVKADCGYLVVPLNGKSHNELEIEWDFVDKVIDFIKIEKRAGFSSKGGSEPFHFNVDDFEDAVVMPSYR
NIDQPQHFYVAEIRHDLNPVSPFPSPELYKTFQDYYTTKYGLMITNTVQPLLDVDHTSARLNLLTPRYMNQKGVALPTSSAETKRARRENLQQKQIL
VPELCDVHVFPASLWRKTVCLPAILYRANYLLLAEELRQKISRETGIGLEGLPERFRFPKLDFGFDTSPEKLLDHEGFSKKNSPNSKISKVEEADVR
LGHPRLKLENCELQAENLKDHQIQPDEIAFTSEDTKTESSSDSDSGVASSSSSSDNCASKFYKCEAQTNLSLEQKSVDKNSSSPSVESSITNYASDD
ITTSTTNSVSCNNQNSTFTKDGCDLSFTPALQQSKSNSSTPAMSVISLSNHAPVTKCTSTISLATSLSPSVSPTFNVDILKTNESTTYLSKDTKSCE
MNQISLVADTSTPGAYCQTGANINATLFLPCSTLTTPPLFMSSSSVLSSSSLSSFCNIKDPSLVEEFSCSPMSTKLVAGKMNPSDPSSSVSPNINAV
LNVKNHLSNSKADLNIPGCEPQGPDAADPALFPATECIHDTLTTSLSNHCASQSPVAQSFNHNHISPSNKAIRQEHDSAHTKNGYLLNGHAKHGNDS
ELSDGPSIIMNTLSEELSGLDIDLIIPQTNTSDTFHTFDTPLQSDTKTNSQCGKKYGQSSWQQAWLNEMEADLLEEENQTDEKSVWNSEEKALTDKE
KNSKNVPEESLLADNEVIKFSFDTQEVKTTQDNSSSQDKNELEKGNSVQVHTKSEEAPTNITDFEIDYWNTADKNTKKSGENSLTKMYNKLIDQDEG
IYDELPSRPLYSKLKELKELSASHQQADAVATVSLDEDRDLTTFVGPSPCLIMQALTMSNANDFFSLERLETIGDSFLKYAITVYLYCSYPGIHEGK
LSYLRSKQVSNYNLYRLGRRKGLAECMVSTKFEPFENWLPPGYVINDDKRRGPVPKVVIVTPGSKVNNSLRNFYLDEADTAKKDEAVLFNKELEWIQ
QSQEADQQDEVQQEPSNNLTPYSLQCHHGLPDKSVADSVEALIGCYLTTCGRTAALIFMSWLGLRVLPKKKKTIEDSNKLERASGSSFCKLAELEFD
ELRCPPSPMFSNTEENQAKLQHLLQGFDSLEEKIGYTFRDKSYLLQAFTHASYHYNTITDCYQRVEFLGDAILDYVITRHLYEDSQKYSPGILTDLR
SALVNNNIFAALAVKWGFHKYFKAISPSLFQVIDKFVKCQKEKKDDDIDIDEEFRELSLEDCEQEEGADEEDEDEEVEMEIPKALGDIFESVAGAIY
LDSGMSLDTVWRVYYRMMKPHIDKYLKSIPKSPVRELLETEPETAKFERPERTMNGKVRVTVNVVGKGVFSGVGRNYRIAKSAAAKKALRSIRTMAQ
NLLG

Figure S1F.

Bgl-Ago2 (*BGLB002396*)

MPPTFLGQHLGNGQPGIPFPFTQLQQDVPPPPVVTTPIGPPEQPPPSFGQPPITPLADFVSPPRPNTGTEGRTISLRANHFQVRVPKGIIHHYEVNI
SPDKCPRRVNREIIDTMVTAYQQKIFQGQKPVFDGRKNLYSREALPIGSEKV**ELEVTLPGEGRDRVF**KVGIRFMGQVSLFALEEALEGRTRQIPMDA
IQALDVVMRHLPSKTYTPVGRSFFSPPEGYDHPLGGGREVWFGFHQSVRPSHWKMMLNIDVSATAFYKEQPVIEFMCEVLDLPDIGEQKRPLADSQR
VKFTKEIKGLKVEITHCGAMRRKYRVCNVTRRPAQTQSFPLQLQSGQTIECTVARYFMERYKMKLQHPHLPCLQVGQEQKHTYLPLEVCNIVGGQRC
IKKLTDMQTSTMIKATARSAPDREKEINNLVAKADFNNDVYLKTFGICVNYDMTELKGRVLPAPKLQYGGRTKAQAVPNQGVWDMRGKQFYQGIEIR
VWAIACFAPQRTVREDALRNFTQQLQRISNDAGMPIMGQPCFCKYATGPDQVEPMFRYLKNTYQGLQLIVVVLPGKTPVYAEVKRVGDICFGLATQC
VQAKNVNKTTPQTLSNLCLKINVKLGGVNSILLPSIRPHVFREPIIFLGADVTHPPAGDTLKPSIAAVVGSMDAHPSRYSATVRVQEHRQEIIRDLA
TMVKELLIQFYRSTRFKPTRIIFYRDGVSEGQFSTVLSHELRAVREACMKLEVDYQPGITFIVVQKRHHTRLFCADRRDQTGRSGNIPAGTTVDQGI
THPTEFDFYLCSHAGIQGTSRPSHYHVLWDDNRFNADELQILTYQLCHTYVRCTRSVSIPAPAYYAHLVAFRARYHLVEKEHDSGEGSRHSDNSEDR
NPLYLARAVTVHPDTCRVMYFA

Figure S1. Amino acid sequences encoded by the candidate genes identified via a standard BLAST search analysis approach of core pieces of protein machinery of the *Biomphalaria glabrata* miRNA pathway. **(A)** *B. glabrata* Drosha (*Bgl*-Drosha) candidate protein identified via BLAST search analysis using the *Hsa*-Drosha sequence as 'bait'. **(B)** *B. glabrata* DGCR8 (*Bgl*-DGCR8) candidate protein identified via BLAST search analysis of the *Hsa*-DGCR8 amino acid sequence. **(C)** *B. glabrata* Exp5 (*Bgl*-Exp5) candidate protein sequence identified via BLAST searching with the *Hsa*-Exp5 sequence. **(D)** Amino acid sequence of the *B. glabrata* Ran (*Bgl*-Ran) candidate protein identified using the *Hsa*-Ran sequence as bait in a standard BLAST search. **(E)** *B. glabrata* Dcr (*Bgl*-Dcr) candidate protein identified via BLAST search analysis using the *Hsa*-Dcr sequence as the search query. **(F)** The amino acid sequence composition of the *B. glabrata* Ago2 (*Bgl*-Ran) protein candidate identified via BLAST search analysis using the sequence of the human Ago2 (*Hsa*-Ago2) protein as the search term. The bolded and underlined text represents the peptide sequence used to generate the antibody specific to the *B. glabrata* Ago2 protein which was used in the Western blot hybridization analyses performed in this study and to determine the tissue-specific localization of *Bgl*-Ago2 in *B. glabrata* whole animal samples post their exposure to the miracidia of the *S. mansoni* parasite.

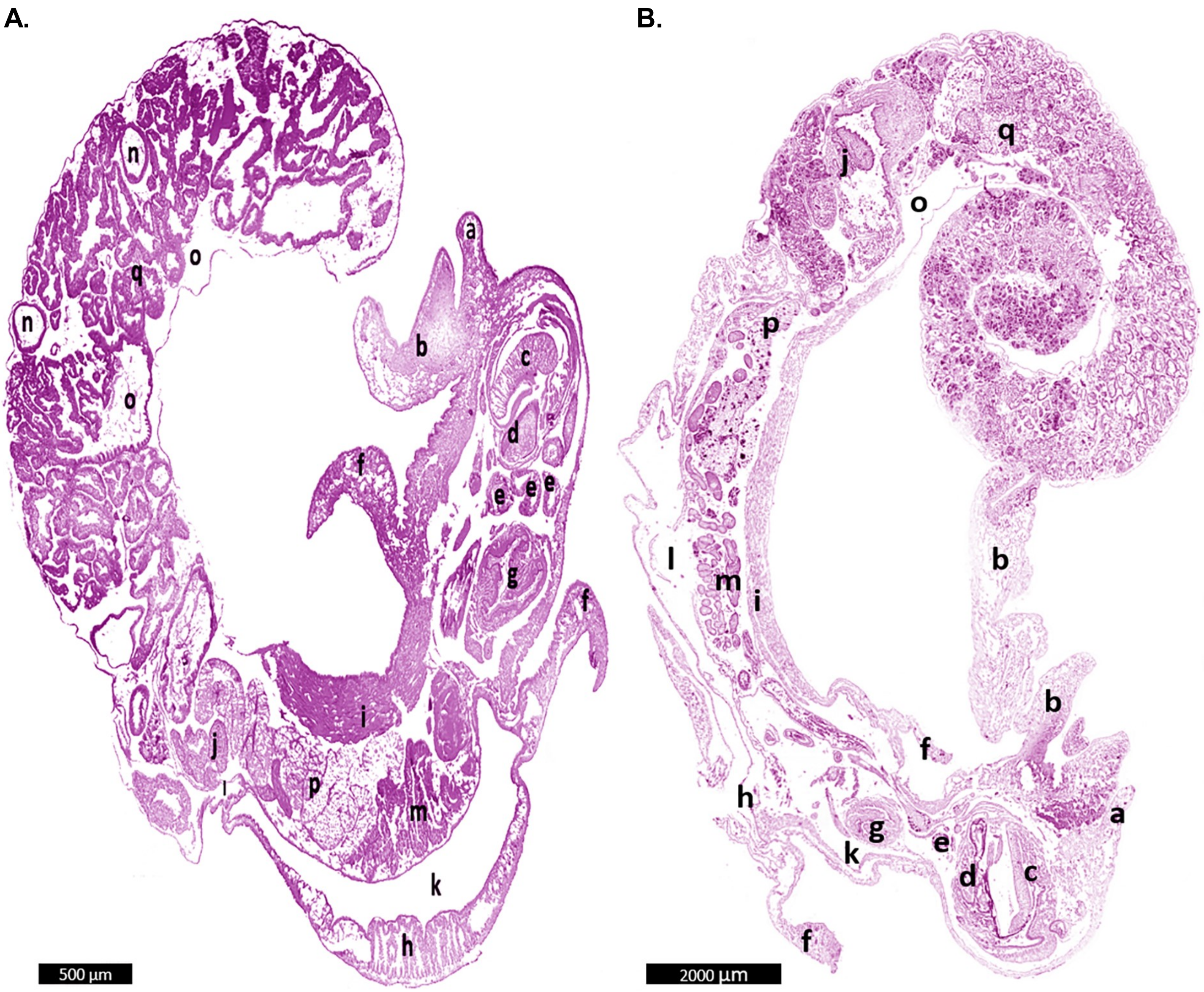

Figure S2. Microscopic analysis of sectioned *Biomphalaria glabrata* whole animals 16 hours and 20 days post their exposure to the miracidia of the *Schistosoma mansoni* parasite. **(A)** and **(B)** anatomical features of *B. glabrata* whole animals (minus their shells) post short and long term exposure to the *S. mansoni* parasite, where (a) = lip, (b) = foot, (c) = buccal mass, (d) = radular sac, (e) = ganglion, (f) = mantle collar, (g) = gonads, (h) = tubular portion of the kidney, (i) = columellar muscle, (j) = albumen gland, (k) = mantle cavity, (l) = pericardial sac, (m) = gizzard, (n) = intestine, (o) = hepatic ducts, (p) = oviduct, and (q) = liver.

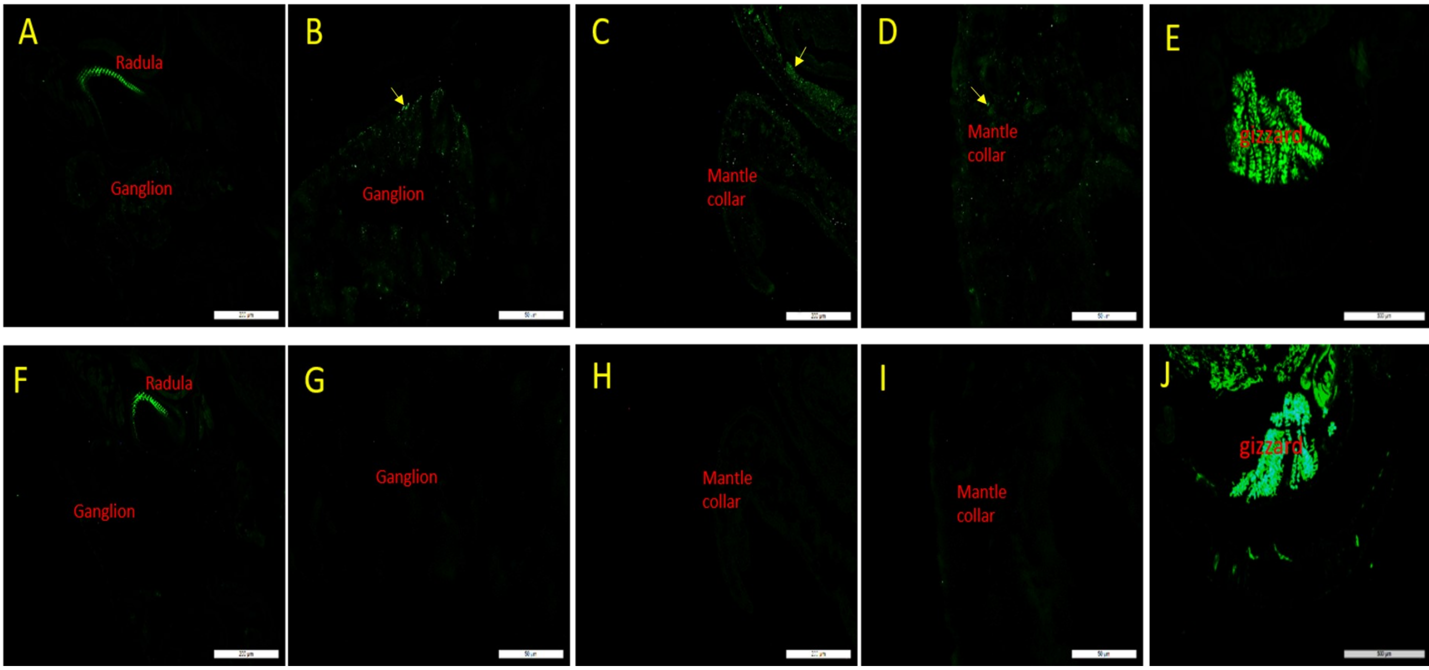

Figure S3. Immunofluroscene assessment of Ago2 protein localization in *Biomphalaria glabrata* animals 16 hours and 20 days post exposure to *Schistosoma mansoni* miracidia. Ago2 protein localization in the **(A)** radula, **(B)** ganglion, **(C)** and **(D)** mantle collar, and **(E)** gizzard of *B. glabrata* animals 16 h post their exposure to *S. mansoni* miracidia. Ago2 protein localization in the **(F)** radula, **(G)** ganglion, **(H)** and **(I)** mantle collar, and gizzard **(J)** of *B. glabrata* animals 20 days post their exposure to *S. mansoni* miracidia.

Table S1. Differentially expressed putative target genes of significantly reduced Biomphalaria glabrata microRNAs

miRNA	Target	Control	16h	log2	p-value
bgl-let-7-5p	BGLB000542	5.2	51.8	2.8	0.0
bgl-let-7-5p	BGLB021546	4.2	28.8	2.4	0.0
bgl-let-7-5p	BGLB002193	6.4	0.4	-4.0	0.0
bgl-let-7-5p	BGLB031061	1.7	18.1	2.9	0.0
bgl-let-7-5p	BGLB014430	1.9	21.1	3.0	0.0
bgl-let-7-5p	BGLB006380	29.7	172.6	2.4	0.0
bgl-let-7-5p	BGLB021769	1.9	13.6	2.3	0.0
bgl-let-7-5p	BGLB018917	106.5	21.8	-2.6	0.0
bgl-let-7-5p	BGLB034922	8.0	57.7	3.0	0.0
bgl-let-7-5p	BGLB029754	0.1	13.2	6.5	0.0
bgl-let-7-5p	BGLB008607	8.9	62.0	2.4	0.0
bgl-let-7-5p	BGLB007725	31.7	191.6	2.6	0.0
bgl-let-7-5p	BGLB035497	3.8	23.2	2.2	0.0
bgl-let-7-5p	BGLB026138	58.6	455.4	3.1	0.0
bgl-let-7-5p	BGLB021329	31.7	170.7	2.2	0.0
bgl-let-7-5p	BGLB040320	2.0	11.5	2.1	0.0
bgl-let-7-5p	BGLB016487	9.5	49.7	2.2	0.0
bgl-let-7-5p	BGLB019257	4.0	20.1	2.0	0.0
bgl-let-7-5p	BGLB012084	2.4	15.8	2.3	0.0
bgl-let-7-5p	BGLB012085	2.7	20.3	2.6	0.0
bgl-let-7-5p	BGLB012086	154.1	22.6	-2.9	0.0
bgl-let-7-5p	BGLB002713	1.0	7.9	2.5	0.0
bgl-let-7-5p	BGLB036266	16.2	2.6	-2.7	0.0
bgl-let-7-5p	BGLB038790	126.0	960.0	2.7	0.0
bgl-let-7-5p	BGLB032491	0.5	6.8	3.5	0.0
bgl-let-7-5p	BGLB028446	2.2	14.2	2.4	0.0
bgl-let-7-5p	BGLB038815	274.1	94.9	-2.0	0.0
bgl-let-7-5p	BGLB019190	4.1	21.4	2.1	0.0
bgl-let-7-5p	BGLB038249	44.9	228.0	2.5	0.0
bgl-let-7-5p	BGLB014050	1.2	78.8	5.7	0.0
bgl-let-7-5p	BGLB034281	1.4	11.3	2.6	0.0
bgl-let-7-5p	BGLB017242	1.8	27.1	3.4	0.0
bgl-let-7-5p	BGLB036017	1.5	11.4	2.9	0.0
bgl-let-7-5p	BGLB026330	257.9	79.9	-2.1	0.0
bgl-let-7-5p	BGLB013940	1.0	14.0	3.5	0.0
bgl-let-7-5p	BGLB004941	0.3	8.8	4.1	0.0
bgl-let-7-5p	BGLB001739	4.2	22.3	2.0	0.0
bgl-let-7-5p	BGLB004090	157.3	756.7	2.3	0.0
bgl-let-7-5p	BGLB007392	7.7	44.1	2.2	0.0
bgl-let-7-5p	BGLB007309	5.3	36.9	2.7	0.0
bgl-let-7-5p	BGLB033668	104.1	38.6	-2.1	0.0
bgl-let-7-5p	BGLB025993	1.0	23.5	4.1	0.0
bgl-let-7-5p	BGLB025439	10.7	60.7	2.5	0.0
bgl-let-7-5p	BGLB033700	1.3	15.8	3.4	0.0
bgl-let-7-5p	BGLB001859	65.1	1190.4	4.4	0.0
bgl-let-7-5p	BGLB001519	3.6	30.5	2.7	0.0
bgl-let-7-5p	BGLB016133	2.3	33.2	4.0	0.0
bgl-let-7-5p	BGLB016973	3.7	26.4	2.3	0.0
bgl-let-7-5p	BGLB002524	479.5	2811.4	2.5	0.0
bgl-let-7-5p	BGLB017799	1.7	17.2	2.8	0.0
bgl-let-7-5p	BGLB002388	7.3	42.4	2.2	0.0
bgl-let-7-5p	BGLB001872	14.7	60.2	2.0	0.0
bgl-let-7-5p	BGLB035593	4.5	30.4	2.2	0.0
bgl-let-7-5p	BGLB039298	17.5	122.0	2.8	0.0
bgl-let-7-5p	BGLB039778	6.3	32.2	2.0	0.0
bgl-let-7-5p	BGLB016561	59.8	17.7	-2.1	0.0
bgl-let-7-5p	BGLB007615	3.9	24.4	2.2	0.0
bgl-let-7-5p	BGLB000172	1.8	11.9	2.5	0.0
bgl-let-7-5p	BGLB000174	2.0	10.7	2.2	0.0
bgl-let-7-5p	BGLB007354	1.7	12.7	2.5	0.0
bgl-let-7-5p	BGLB008049	4.0	19.9	2.1	0.0
bgl-let-7-5p	BGLB003188	24.4	7.3	-2.2	0.0
bgl-let-7-5p	BGLB000955	4.8	29.8	2.3	0.0
bgl-let-7-5p	BGLB001617	1.1	9.7	2.6	0.0

bgl-let-7-5p	BGLB008404	41.6	219.6	2.7	0.0
bgl-let-7-5p	BGLB039131	1.1	7.7	2.5	0.0
bgl-let-7-5p	BGLB020679	558.7	189.9	-2.1	0.0
bgl-let-7-5p	BGLB016382	0.4	4.4	3.4	0.0
bgl-let-7-5p	BGLB023364	1.0	18.7	3.9	0.0
bgl-let-7-5p	BGLB008486	2.7	15.0	2.1	0.0
bgl-let-7-5p	BGLB039416	49.1	228.5	2.1	0.0
bgl-let-7-5p	BGLB027524	20.1	0.8	-5.1	0.0
bgl-let-7-5p	BGLB002851	3.1	17.9	2.1	0.0
bgl-let-7-5p	BGLB032070	1.8	18.8	2.9	0.0
bgl-let-7-5p	BGLB028389	1.7	33.8	3.8	0.0
bgl-let-7-5p	BGLB036283	0.4	7.4	3.8	0.0
bgl-let-7-5p	BGLB002420	3.2	30.5	2.8	0.0
bgl-let-7-5p	BGLB003191	3.3	26.4	2.5	0.0
bgl-let-7-5p	BGLB020494	39.3	351.6	3.3	0.0
bgl-let-7-5p	BGLB022661	2.6	26.2	2.9	0.0
bgl-let-7-5p	BGLB039391	5.4	29.5	2.2	0.0
bgl-let-7-5p	BGLB031994	4.7	36.1	2.5	0.0
bgl-let-7-5p	BGLB010820	1.5	11.1	2.5	0.0
bgl-let-7-5p	BGLB010990	21.3	5.0	-2.1	0.0
bgl-let-7-5p	BGLB035665	2.0	14.6	2.8	0.0
bgl-let-7-5p	BGLB018346	0.2	8.8	4.7	0.0
bgl-let-7-5p	BGLB012596	5.4	30.2	2.2	0.0
bgl-let-7-5p	BGLB030706	18.6	89.7	2.0	0.0
bgl-let-7-5p	BGLB027999	3.4	17.8	2.0	0.0
bgl-let-7-5p	BGLB012995	5.7	49.8	2.9	0.0
bgl-let-7-5p	BGLB031226	1.7	20.2	3.2	0.0
bgl-let-7-5p	BGLB012174	11.8	62.9	2.5	0.0
bgl-let-7-5p	BGLB003002	3.3	52.2	3.6	0.0
bgl-let-7-5p	BGLB037003	33.6	248.1	2.5	0.0
bgl-let-7-5p	BGLB030987	0.0	21.8	7.9	0.0
bgl-let-7-5p	BGLB004463	3.1	26.2	2.8	0.0
bgl-let-7-5p	BGLB012984	4.6	23.8	2.1	0.0
bgl-let-7-5p	BGLB016513	3.8	22.9	2.4	0.0
bgl-let-7-5p	BGLB013485	3.4	18.1	2.1	0.0
bgl-let-7-5p	BGLB027538	5.1	59.2	3.7	0.0
bgl-let-7-5p	BGLB014112	4.0	30.8	2.5	0.0
bgl-let-7-5p	BGLB019764	11.2	69.3	2.3	0.0
bgl-let-7-5p	BGLB013655	13.6	157.1	3.6	0.0
bgl-let-7-5p	BGLB013171	1.8	13.8	2.5	0.0
bgl-let-7-5p	BGLB014205	5.2	29.7	2.1	0.0
bgl-let-7-5p	BGLB010384	2.2	14.7	2.4	0.0
bgl-let-7-5p	BGLB036286	4.2	131.6	4.7	0.0
bgl-let-7-5p	BGLB017299	2.3	16.0	2.3	0.0
bgl-let-7-5p	BGLB038342	2.1	28.0	3.7	0.0
bgl-let-7-5p	BGLB022176	3.9	26.6	2.5	0.0
bgl-let-7-5p	BGLB005339	3.6	31.8	2.7	0.0
bgl-let-7-5p	BGLB022137	259.6	36.3	-3.2	0.0
bgl-let-7-5p	BGLB002219	0.5	8.6	3.8	0.0
bgl-let-7-5p	BGLB008240	1.6	11.5	2.5	0.0
bgl-let-7-5p	BGLB005603	3.7	25.4	2.3	0.0
bgl-let-7-5p	BGLB035966	3.8	31.3	3.0	0.0
bgl-let-7-5p	BGLB022543	9.5	77.1	3.0	0.0
bgl-let-7-5p	BGLB006719	25.0	169.6	2.4	0.0
bgl-let-7-5p	BGLB040298	1.3	9.2	2.5	0.0
bgl-let-7-5p	BGLB009540	30.9	179.8	2.0	0.0
bgl-let-7-5p	BGLB025668	238.8	60.3	-2.1	0.0
bgl-let-7-5p	BGLB009156	2.0	34.8	3.7	0.0
bgl-let-7-5p	BGLB038609	8.4	39.3	2.0	0.0
bgl-let-7-5p	BGLB006482	2.3	12.7	2.1	0.0
bgl-let-7-5p	BGLB008073	33.4	491.4	4.2	0.0
bgl-let-7-5p	BGLB009806	0.8	15.1	3.7	0.0
bgl-let-7-5p	BGLB036055	4.4	21.6	2.0	0.0
bgl-let-7-5p	BGLB029609	10.8	104.8	3.3	0.0
bgl-let-7-5p	BGLB021015	36.4	289.8	2.9	0.0
bgl-let-7-5p	BGLB036752	2.2	11.6	2.1	0.0

bgl-let-7-5p	BGLB007648	10.8	64.9	2.4	0.0
bgl-let-7-5p	BGLB010356	0.4	11.8	4.6	0.0
bgl-let-7-5p	BGLB011132	1.5	10.6	2.6	0.0
bgl-let-7-5p	BGLB029341	1.4	11.2	2.9	0.0
bgl-let-7-5p	BGLB019356	120.9	38.8	-2.0	0.0
bgl-let-7-5p	BGLB021427	1.9	27.8	3.9	0.0
bgl-let-7-5p	BGLB009575	4.0	23.8	2.2	0.0
bgl-let-7-5p	BGLB033791	19.4	96.5	2.4	0.0
bgl-let-7-5p	BGLB016238	1.9	17.6	2.7	0.0
bgl-let-7-5p	BGLB011861	17.3	4.2	-2.5	0.0
bgl-let-7-5p	BGLB024088	2.2	17.6	2.6	0.0
bgl-let-7-5p	BGLB040370	7.4	36.3	2.1	0.0
bgl-let-7-5p	BGLB028284	6.0	42.5	2.5	0.0
bgl-let-7-5p	BGLB016714	8.3	197.0	4.8	0.0
bgl-let-7-5p	BGLB024002	3.6	61.6	4.2	0.0
bgl-let-7-5p	BGLB039138	7.3	41.5	2.2	0.0
bgl-let-7-5p	BGLB022486	4.9	31.3	2.3	0.0
bgl-let-7-5p	BGLB037676	15.4	0.0	-7.6	0.0
bgl-let-7-5p	BGLB010948	2.3	30.4	3.4	0.0
bgl-let-7-5p	BGLB027454	319.6	77.4	-2.6	0.0
bgl-let-7-5p	BGLB030630	0.6	27.8	4.9	0.0
bgl-let-7-5p	BGLB012894	1.7	17.3	3.2	0.0
bgl-let-7-5p	BGLB028646	20.7	5.8	-2.2	0.0
bgl-let-7-5p	BGLB028855	16.9	78.6	2.0	0.0
bgl-let-7-5p	BGLB013720	7.7	43.4	2.2	0.0
bgl-let-7-5p	BGLB032707	39.6	4.8	-3.4	0.0
bgl-let-7-5p	BGLB039634	9.4	0.3	-4.7	0.0
bgl-let-7-5p	BGLB029149	68.6	21.3	-2.0	0.0
bgl-miR-216a-5p	BGLB000542	5.2	51.8	2.8	0.0
bgl-miR-216a-5p	BGLB002193	6.4	0.4	-4.0	0.0
bgl-miR-216a-5p	BGLB006380	29.7	172.6	2.4	0.0
bgl-miR-216a-5p	BGLB018917	106.5	21.8	-2.6	0.0
bgl-miR-216a-5p	BGLB034922	8.0	57.7	3.0	0.0
bgl-miR-216a-5p	BGLB040320	2.0	11.5	2.1	0.0
bgl-miR-216a-5p	BGLB032491	0.5	6.8	3.5	0.0
bgl-miR-216a-5p	BGLB013940	1.0	14.0	3.5	0.0
bgl-miR-216a-5p	BGLB004941	0.3	8.8	4.1	0.0
bgl-miR-216a-5p	BGLB001739	4.2	22.3	2.0	0.0
bgl-miR-216a-5p	BGLB006441	2.1	16.9	2.6	0.0
bgl-miR-216a-5p	BGLB025993	1.0	23.5	4.1	0.0
bgl-miR-216a-5p	BGLB001519	3.6	30.5	2.7	0.0
bgl-miR-216a-5p	BGLB001872	14.7	60.2	2.0	0.0
bgl-miR-216a-5p	BGLB035593	4.5	30.4	2.2	0.0
bgl-miR-216a-5p	BGLB007615	3.9	24.4	2.2	0.0
bgl-miR-216a-5p	BGLB000174	2.0	10.7	2.2	0.0
bgl-miR-216a-5p	BGLB000955	4.8	29.8	2.3	0.0
bgl-miR-216a-5p	BGLB027524	20.1	0.8	-5.1	0.0
bgl-miR-216a-5p	BGLB002851	3.1	17.9	2.1	0.0
bgl-miR-216a-5p	BGLB003191	3.3	26.4	2.5	0.0
bgl-miR-216a-5p	BGLB010820	1.5	11.1	2.5	0.0
bgl-miR-216a-5p	BGLB025042	1.5	20.2	3.4	0.0
bgl-miR-216a-5p	BGLB012596	5.4	30.2	2.2	0.0
bgl-miR-216a-5p	BGLB014112	4.0	30.8	2.5	0.0
bgl-miR-216a-5p	BGLB017299	2.3	16.0	2.3	0.0
bgl-miR-216a-5p	BGLB002219	0.5	8.6	3.8	0.0
bgl-miR-216a-5p	BGLB024618	0.8	26.6	5.0	0.0
bgl-miR-216a-5p	BGLB005874	0.7	10.6	3.3	0.0
bgl-miR-216a-5p	BGLB009156	2.0	34.8	3.7	0.0
bgl-miR-216a-5p	BGLB006482	2.3	12.7	2.1	0.0
bgl-miR-216a-5p	BGLB011132	1.5	10.6	2.6	0.0
bgl-miR-216a-5p	BGLB029341	1.4	11.2	2.9	0.0
bgl-miR-216a-5p	BGLB009575	4.0	23.8	2.2	0.0
bgl-miR-216a-5p	BGLB011861	17.3	4.2	-2.5	0.0
bgl-miR-216a-5p	BGLB022201	0.2	8.7	4.5	0.0
bgl-miR-216a-5p	BGLB028284	6.0	42.5	2.5	0.0
bgl-miR-216a-5p	BGLB022486	4.9	31.3	2.3	0.0

bgl-miR-216a-5p	BGLB028646	20.7	5.8	-2.2	0.0
bgl-miR-216a-5p	BGLB039634	9.4	0.3	-4.7	0.0
bgl-miR-252a-5p	BGLB000542	5.2	51.8	2.8	0.0
bgl-miR-252a-5p	BGLB026138	58.6	455.4	3.1	0.0
bgl-miR-252a-5p	BGLB040320	2.0	11.5	2.1	0.0
bgl-miR-252a-5p	BGLB034281	1.4	11.3	2.6	0.0
bgl-miR-252a-5p	BGLB025993	1.0	23.5	4.1	0.0
bgl-miR-252a-5p	BGLB002388	7.3	42.4	2.2	0.0
bgl-miR-252a-5p	BGLB000955	4.8	29.8	2.3	0.0
bgl-miR-252a-5p	BGLB020679	558.7	189.9	-2.1	0.0
bgl-miR-252a-5p	BGLB008486	2.7	15.0	2.1	0.0
bgl-miR-252a-5p	BGLB027524	20.1	0.8	-5.1	0.0
bgl-miR-252a-5p	BGLB003191	3.3	26.4	2.5	0.0
bgl-miR-252a-5p	BGLB012995	5.7	49.8	2.9	0.0
bgl-miR-252a-5p	BGLB031226	1.7	20.2	3.2	0.0
bgl-miR-252a-5p	BGLB012984	4.6	23.8	2.1	0.0
bgl-miR-252a-5p	BGLB008240	1.6	11.5	2.5	0.0
bgl-miR-252a-5p	BGLB009540	30.9	179.8	2.0	0.0
bgl-miR-252a-5p	BGLB008073	33.4	491.4	4.2	0.0
bgl-miR-252a-5p	BGLB009806	0.8	15.1	3.7	0.0
bgl-miR-252a-5p	BGLB010356	0.4	11.8	4.6	0.0
bgl-miR-252a-5p	BGLB032367	13.1	240.1	3.9	0.0
bgl-miR-252a-5p	BGLB022486	4.9	31.3	2.3	0.0
bgl-miR-252a-5p	BGLB012894	1.7	17.3	3.2	0.0
bgl-miR-281-5p	BGLB000542	5.2	51.8	2.8	0.0
bgl-miR-281-5p	BGLB021546	4.2	28.8	2.4	0.0
bgl-miR-281-5p	BGLB031061	1.7	18.1	2.9	0.0
bgl-miR-281-5p	BGLB006380	29.7	172.6	2.4	0.0
bgl-miR-281-5p	BGLB034922	8.0	57.7	3.0	0.0
bgl-miR-281-5p	BGLB008607	8.9	62.0	2.4	0.0
bgl-miR-281-5p	BGLB026138	58.6	455.4	3.1	0.0
bgl-miR-281-5p	BGLB002713	1.0	7.9	2.5	0.0
bgl-miR-281-5p	BGLB001739	4.2	22.3	2.0	0.0
bgl-miR-281-5p	BGLB025993	1.0	23.5	4.1	0.0
bgl-miR-281-5p	BGLB002524	479.5	2811.4	2.5	0.0
bgl-miR-281-5p	BGLB002388	7.3	42.4	2.2	0.0
bgl-miR-281-5p	BGLB039298	17.5	122.0	2.8	0.0
bgl-miR-281-5p	BGLB007615	3.9	24.4	2.2	0.0
bgl-miR-281-5p	BGLB001617	1.1	9.7	2.6	0.0
bgl-miR-281-5p	BGLB039416	49.1	228.5	2.1	0.0
bgl-miR-281-5p	BGLB036283	0.4	7.4	3.8	0.0
bgl-miR-281-5p	BGLB003191	3.3	26.4	2.5	0.0
bgl-miR-281-5p	BGLB031226	1.7	20.2	3.2	0.0
bgl-miR-281-5p	BGLB037003	33.6	248.1	2.5	0.0
bgl-miR-281-5p	BGLB030987	0.0	21.8	7.9	0.0
bgl-miR-281-5p	BGLB012984	4.6	23.8	2.1	0.0
bgl-miR-281-5p	BGLB016513	3.8	22.9	2.4	0.0
bgl-miR-281-5p	BGLB020273	1.1	16.0	3.3	0.0
bgl-miR-281-5p	BGLB010384	2.2	14.7	2.4	0.0
bgl-miR-281-5p	BGLB038342	2.1	28.0	3.7	0.0
bgl-miR-281-5p	BGLB034003	14.6	179.2	3.2	0.0
bgl-miR-281-5p	BGLB009540	30.9	179.8	2.0	0.0
bgl-miR-281-5p	BGLB008073	33.4	491.4	4.2	0.0
bgl-miR-281-5p	BGLB007648	10.8	64.9	2.4	0.0
bgl-miR-281-5p	BGLB032367	13.1	240.1	3.9	0.0
bgl-miR-281-5p	BGLB021427	1.9	27.8	3.9	0.0
bgl-miR-281-5p	BGLB037676	15.4	0.0	-7.6	0.0
bgl-miR-281-5p	BGLB036743	1.1	63.6	5.7	0.0
bgl-miR-2a-2-3p	BGLB031061	1.7	18.1	2.9	0.0
bgl-miR-2a-2-3p	BGLB014430	1.9	21.1	3.0	0.0
bgl-miR-2a-2-3p	BGLB029603	2.9	24.6	3.2	0.0
bgl-miR-2a-2-3p	BGLB029754	0.1	13.2	6.5	0.0
bgl-miR-2a-2-3p	BGLB026138	58.6	455.4	3.1	0.0
bgl-miR-2a-2-3p	BGLB012085	2.7	20.3	2.6	0.0
bgl-miR-2a-2-3p	BGLB002713	1.0	7.9	2.5	0.0
bgl-miR-2a-2-3p	BGLB036266	16.2	2.6	-2.7	0.0

bgl-miR-2a-2-3p	BGLB010765	9.4	56.3	2.4	0.0
bgl-miR-2a-2-3p	BGLB017398	46.0	338.9	3.0	0.0
bgl-miR-2a-2-3p	BGLB019190	4.1	21.4	2.1	0.0
bgl-miR-2a-2-3p	BGLB038249	44.9	228.0	2.5	0.0
bgl-miR-2a-2-3p	BGLB034281	1.4	11.3	2.6	0.0
bgl-miR-2a-2-3p	BGLB026330	257.9	79.9	-2.1	0.0
bgl-miR-2a-2-3p	BGLB001739	4.2	22.3	2.0	0.0
bgl-miR-2a-2-3p	BGLB033700	1.3	15.8	3.4	0.0
bgl-miR-2a-2-3p	BGLB001859	65.1	1190.4	4.4	0.0
bgl-miR-2a-2-3p	BGLB001519	3.6	30.5	2.7	0.0
bgl-miR-2a-2-3p	BGLB016133	2.3	33.2	4.0	0.0
bgl-miR-2a-2-3p	BGLB016973	3.7	26.4	2.3	0.0
bgl-miR-2a-2-3p	BGLB006271	0.3	5.7	3.5	0.0
bgl-miR-2a-2-3p	BGLB017799	1.7	17.2	2.8	0.0
bgl-miR-2a-2-3p	BGLB008049	4.0	19.9	2.1	0.0
bgl-miR-2a-2-3p	BGLB000955	4.8	29.8	2.3	0.0
bgl-miR-2a-2-3p	BGLB028389	1.7	33.8	3.8	0.0
bgl-miR-2a-2-3p	BGLB002420	3.2	30.5	2.8	0.0
bgl-miR-2a-2-3p	BGLB003191	3.3	26.4	2.5	0.0
bgl-miR-2a-2-3p	BGLB039391	5.4	29.5	2.2	0.0
bgl-miR-2a-2-3p	BGLB010593	5.1	26.2	2.2	0.0
bgl-miR-2a-2-3p	BGLB012596	5.4	30.2	2.2	0.0
bgl-miR-2a-2-3p	BGLB012984	4.6	23.8	2.1	0.0
bgl-miR-2a-2-3p	BGLB014112	4.0	30.8	2.5	0.0
bgl-miR-2a-2-3p	BGLB013171	1.8	13.8	2.5	0.0
bgl-miR-2a-2-3p	BGLB010384	2.2	14.7	2.4	0.0
bgl-miR-2a-2-3p	BGLB038342	2.1	28.0	3.7	0.0
bgl-miR-2a-2-3p	BGLB022137	259.6	36.3	-3.2	0.0
bgl-miR-2a-2-3p	BGLB024618	0.8	26.6	5.0	0.0
bgl-miR-2a-2-3p	BGLB040298	1.3	9.2	2.5	0.0
bgl-miR-2a-2-3p	BGLB038609	8.4	39.3	2.0	0.0
bgl-miR-2a-2-3p	BGLB006482	2.3	12.7	2.1	0.0
bgl-miR-2a-2-3p	BGLB008073	33.4	491.4	4.2	0.0
bgl-miR-2a-2-3p	BGLB009806	0.8	15.1	3.7	0.0
bgl-miR-2a-2-3p	BGLB029609	10.8	104.8	3.3	0.0
bgl-miR-2a-2-3p	BGLB021015	36.4	289.8	2.9	0.0
bgl-miR-2a-2-3p	BGLB024002	3.6	61.6	4.2	0.0
bgl-miR-2a-2-3p	BGLB039138	7.3	41.5	2.2	0.0
bgl-miR-2a-2-3p	BGLB027454	319.6	77.4	-2.6	0.0
bgl-miR-2a-2-3p	BGLB039634	9.4	0.3	-4.7	0.0
bgl-miR-2a-2-3p	BGLB029149	68.6	21.3	-2.0	0.0
